# Synthesis Strategies and Electrochemical Research Progress of Nano/Microscale Metal–Organic Frameworks

**DOI:** 10.1002/smsc.202200042

**Published:** 2022-10-17

**Authors:** Shixian Wang, Wenhui Hu, Yue Ru, Yuxin Shi, Xiaotian Guo, Yangyang Sun, Huan Pang

**Affiliations:** ^1^ School of Chemistry and Chemical Engineering Yangzhou University Yangzhou Jiangsu 225009 P. R. China

**Keywords:** energy conversion, energy storage, nano/microscale metal–organic frameworks

## Abstract

Nanoscale/microscale metal–organic frameworks (nano/microscale MOFs) are considered kinds of nanomaterials with profound application potentials in many fields due to the high specific surface area, permanent porosity, and multiple chemical functions. This review focuses on the specific synthesis strategies of nano/microscale MOFs, such as controlled mediation, template, one‐pot, and interface growth methods, through which the shape and size of the crystal can be regulated during the nucleation process. After these targeted design and synthesis strategies, nano/microscale MOFs are optimized for energy storage, catalysis, and biomedical applications based on several merits, including a large specific surface area with more active sites, smaller ion transfer resistance, and structural stability. In addition, challenges and prospects of nano/microscale MOF materials are summarized for advanced energy storage and conversion applications.

## Graphical abstract

Nano/microscale metal–organic frameworks (MOFs) with controllable size and shape can be synthesized by various strategies, which possess unique properties compared with bulk MOFs and show great potential in energy storage and conversion fields.

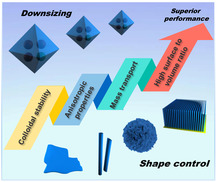

## 1 Introduction

In the past few decades, the remarkable development in nanoscience and nanotechnology has provided the possibility for the structural design of rational nanomaterial.^[^ ^1^, ^2^, ^3^, ^4^, ^5^, ^6^ ^]^ More importantly, nanomaterials possess unique surface effects, bulk effects, quantum size effects, and macroscopic tunneling effects and exhibit many distinct physicochemical properties that are different from other materials and individual molecules.^[^ ^7^, ^8^, ^9^ ^]^ Due to their excellent physical and chemical properties, they have been widely applied in many fields, such as energy storage and conversion, life science, and medicine. However, the obvious point, planar, and line defects in nanomaterials lead to the formation of a large number of vacancies and crystal distortions in crystals, which is an urgent challenge for the future development of expanded nanomaterials.

Metal–organic frameworks (MOFs), as porous crystalline materials, are composed of coordinated bonds and metal nodes. In recent years, MOFs have been widely studied by researchers due to their unique structural characteristics, such as high specific surface area, controllable shape and size, and uniform internal pore size distribution.^[^ ^10^, ^11^, ^12^, ^13^, ^14^, ^15^, ^16^ ^]^ As a result, they have been used in many applications, such as energy storage (batteries, supercapacitor), catalysis, and biomedical applications, where its uniformly distributed pore size allows for faster ion transport, and in catalysis, where it is synthesized into ultrathin flakes for improved catalytic performance due to its controlled morphology.^[^ ^16^, ^17^, ^18^, ^19^, ^20^, ^21^, ^22^, ^23^ ^]^ While the advantages of MOF have been recognized by researchers, there are still many issues that havwe to be refined and optimized. For energy storage and conversion, the electrical conductivity of MOF materials is poor, and the original structure cannot be maintained after a long time of charge and discharge, which may result in structural collapse and further influence the performance.^[^ ^24^, ^25^, ^26^, ^27^ ^]^ Some active sites in large‐diameter MOFs are not sufficiently exposed, resulting in poor catalytic performance. Therefore, the structural design and performance optimization of MOF materials are still challenging.^[^ ^28^, ^29^, ^30^, ^31^ ^]^

**Figure 1:**
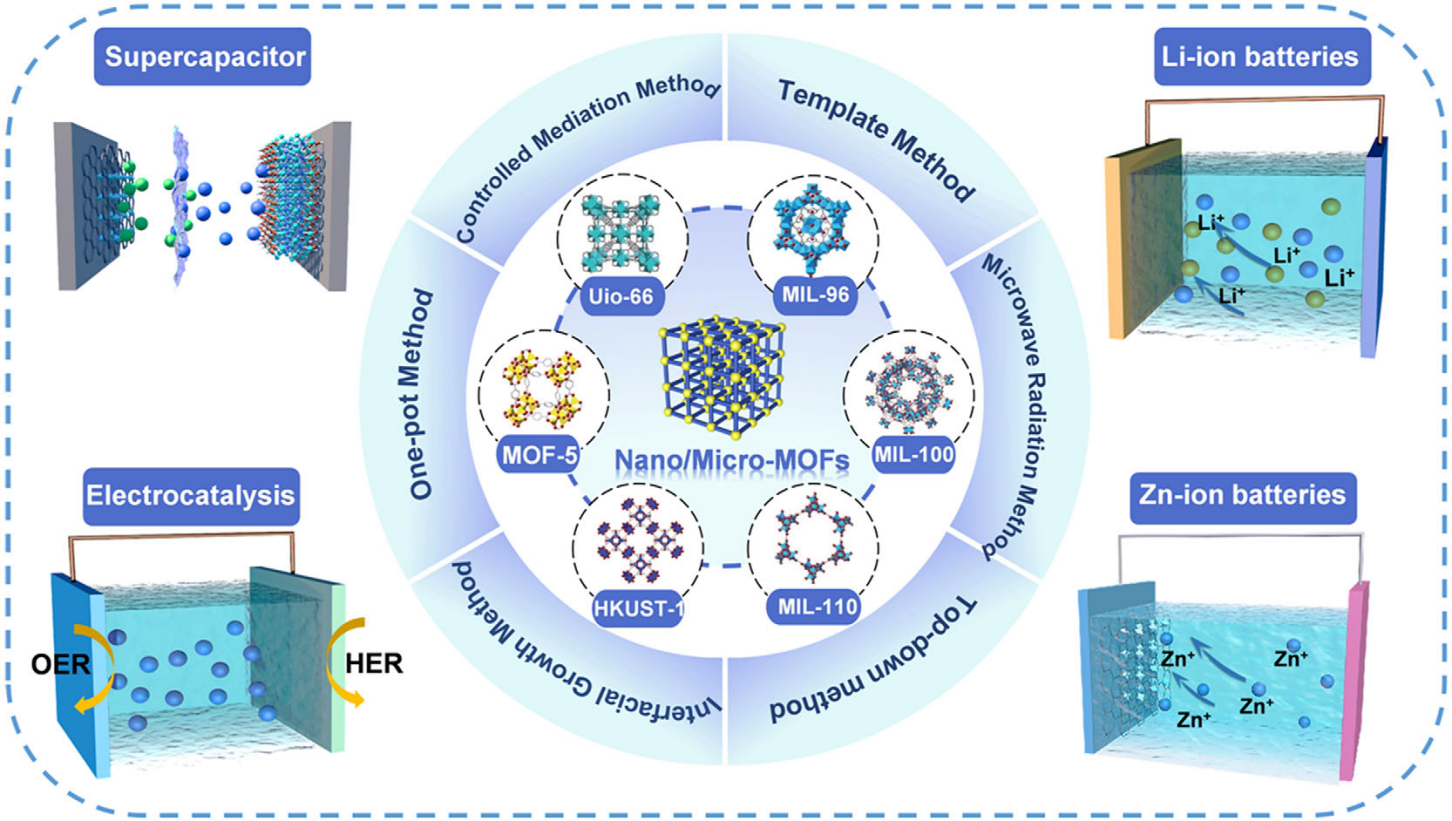
Schematic diagram of preparation and electrochemical application of nano/microscale MOFs. Reproduced with permission.^[^ ^53^ ^]^ Copyright 2018, Royal Society of Chemistry.

**Figure 2:**
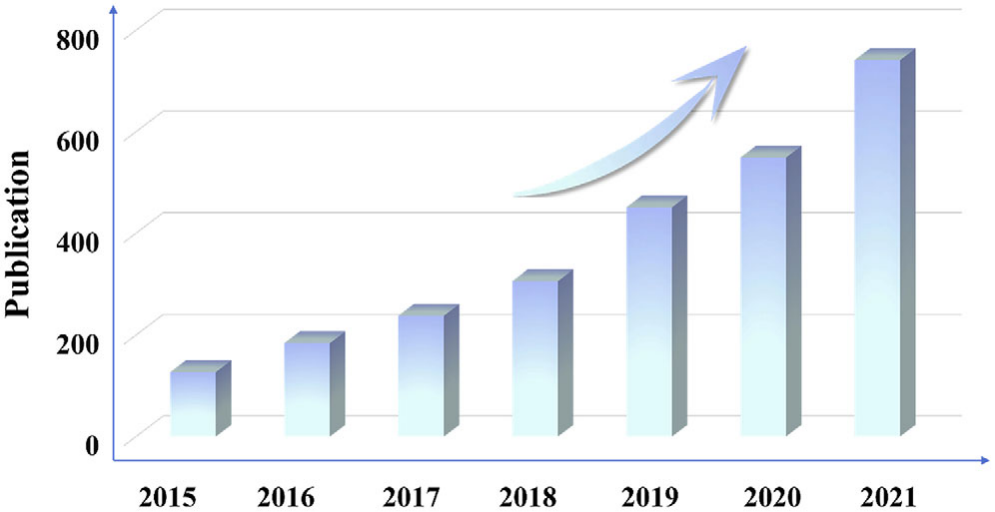
Summary of publications related to the application of nano/micro MOF materials in recent years (2015–2021), as searched in “Web of Science”.

Due to the poor conductivity and structural stability of large‐sized MOFs, researchers have tried to reduce the particle size of MOF materials to improve the electrical energy storage and electrocatalysis performance (**Figure** **1**). Studies have confirmed that nano/microscale MOFs have more flexible and controllable shape and size, higher porosity, and larger specific surface area than traditional MOF materials.^[^ ^32^, ^33^, ^34^, ^35^, ^36^, ^37^, ^38^, ^39^, ^40^, ^41^ ^]^ Therefore, some specific synthesis methods, such as template, top‐down, and interface growth method, are widely used to synthesize nano/microscale MOFs with various morphologies.^[^ ^17^, ^42^, ^43^, ^44^ ^]^ Moreover, the methods mentioned can effectively control and inhibit crystal nucleation and growth in the preparation process without further differentiation after synthesis.^[^ ^45^, ^46^ ^]^ The prepared nano/microscale MOFs not only possess high catalytic properties but are also able to maintain structural integrity and provide sufficient space for ion transfer after multiple charge/discharge cycles to improve their electrical conductivity. Therefore, nano/microscale MOFs are obviously superior to other similar materials in terms of electrochemical performance for energy storage/conversion application and the corresponding publications in recent years have been summarized in **Figure** **2**.^[^ ^47^, ^48^, ^49^, ^50^, ^51^ ^]^ Because the controllable aperture can provide a good opportunity for drug delivery, MOFs have been widely used in biology and pharmaceutical applications.^[^ ^52^ ^]^ In addition, some nano/microscale MOFs can be assembled into other complex structures, which allow previously dispersed particles to be assembled together to produce new chemical and physical properties.

In this review, various synthesis methods such as controlled mediation, template, one‐pot, and top‐down methods are introduced by which the size and shape of nano/microscale MOFs can be effectively controlled and tuned. After targeted design, the nano/microscale MOFs, with high surface‐to‐volume ratio, anisotropic properties, and a large number of ion transport channels, were prepared and widely applied in various fields (energy storage/conversion systems, biological, and pharmaceutical applications). For energy storage systems, these characteristics can make MOFs maintain the original shape after multiple charge and discharge cycles without affecting the conductivity of the material due to structural collapse. For energy conversion systems, the porosity of MOF materials allows exposure of a large number of active sites, improving the catalytic capacity of the materials. In addition, nano/microscale MOFs have also shown great promise in biological and pharmaceutical applications as MOFs can be evenly decorated on the surface of MOFs nanoparticles to cap the pores in a convenient manner, leaving out cumbersome and time‐consuming anchoring procedures for other gatekeepers. Unlike other reviews, which are written from the perspective of synthesis, this review focuses on the improvement of nano/microscale MOF material properties and creatively summarizes the recent research progress in energy storage/conversion systems, biological, and pharmaceutical applications. Finally, we also point out the challenges and prospects of the research subjects and put forward our own insights into the future research direction.

## 2 Synthesis Strategy

In recent years, the synthesis of micro/nano‐MOF materials, which are made up with metal ions (Fe, Co, Ni, Fe, Zn) and organic ligands, has become a research hotpot. Many synthesis strategies have been applied in the synthesis of MOFs, such as controlled mediation, template, and one‐pot methods.^[^ ^54^, ^55^, ^56^, ^57^ ^]^ Controlled mediation method can change the morphology of MOFs by adding regulators. Similarly, in addition to adjusting MOF itself, changes in the growth environment of MOF are also explored. For example, template method uses templates with different pore sizes to obtain MOF crystals having different sizes and morphologies Hence, the interfacial growth method can use its own physical properties to obtain ultrathin nanosheet structures. More significantly, one‐pot method could be applied to obtain complex nanocrystal structures in one step. The nano/microscale MOFs obtained by the methods mentioned above have been proved to have good application potentials in electrochemical properties. For example, ultrathin MOF nanosheets increase the exposed specific surface area and structural porosity, thus reducing the mass transfer limitation, and have better conductivity and electrocatalytic stability than ordinary MOF nanosheets. Nanoflowers have more active surface centers and better electron transport capacity, which is of great significance to improve the performance of energy storage devices and the catalytic capacity of catalysts.

### 2.1 Controlled Mediation Method

Researchers have been committed to the preparation of nano/microscale MOFs with various morphologies, and the controlled mediation method can effectively solve this problem. This method usually refers to the adjustment of some material intermediaries in the synthesis process, such as the concentration and ratio of the reaction solvent or the pressure of the gas during the synthesis process, and finally obtaining the optimal size and structure of the material.^[^ ^58^, ^59^, ^60^ ^]^ As a widely used material in recent years, the original shape and size of MOF can no longer meet the needs of today's applications.^[^ ^61^, ^62^ ^]^ Researchers have found that the method has a high success rate and has been extensively investigated. This method can be applied to the micronization of various MOFs, which can eventually lead to smaller MOF sizes or desired nano/microscale MOF morphologies such as nanowires and nanorods. This experiment confirmed that acetic acid, as a regulator, could inhibit the binding of metal ions to organic ligands and limit the growth of MOFs, resulting in nanorods. In the controlled mediation method, the structure and size of MOF can be controlled not only by adjusting the acetic acid but also by adjusting the content of solvent and water involved in the reaction.

**Figure 3:**
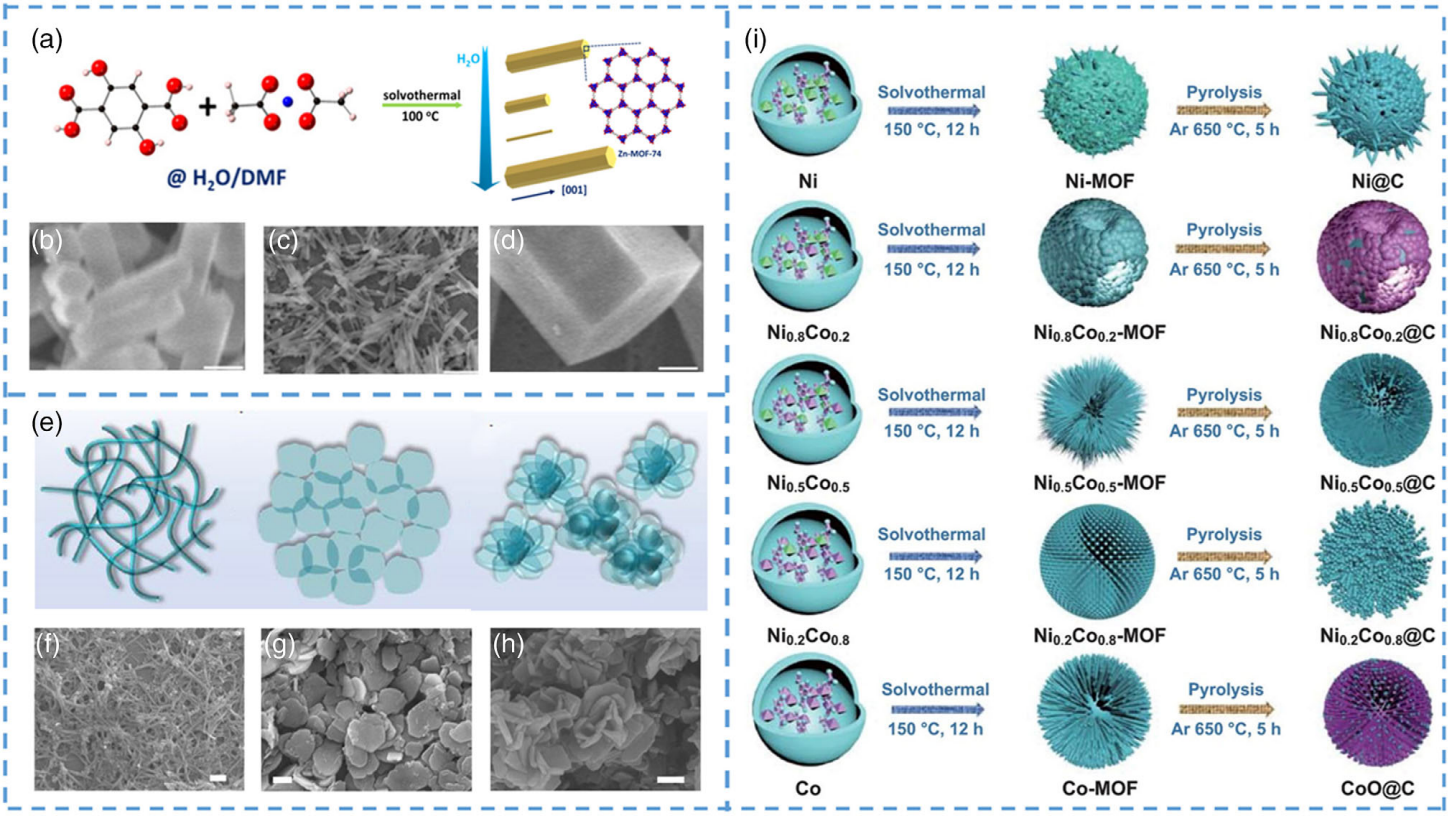
a) Diagram of the synthesis of ZRods. The size of the synthetic crystal can be adjusted by changing the ratio of H_2_O to DMF. b–d) SEM images of ZRods synthesized with an H_2_O/DMF volume ratios of 0 (b), 0.05 (c), and 0.167 (d) (scale bar: 500 nm). e) The morphological transformation process of MOF nanomaterials from 1D nanofibers to 3D aggregates. f–h) SEM images (scale bars: 200 nm). i) Schematic diagram of preparation of precursor Ni_1−*x*_Co_*x*_. a–d) Reproduced with permission.^[^ ^64^ ^]^ Copyright 2021, Elsevier. e–h) Reproduced with permission.^[^ ^65^ ^]^ Copyright 2021, Published by Oxford University Press on behalf of China Science Publishing & Media Ltd. i) Reproduced with permission.^[^ ^66^ ^]^ Copyright 2020, Springer.

Capping agents are widely used in the regulation process to limit particle growth to the maximum extent, act on particle surface reactions, and do not enter the particle interior. The best capping agents, usually those with only one functional monomer, are able to form new coordination bonds with metal ions in the MOFs. Nucleation is induced by adding a solvent with the same function as the organic ligand to prevent the interaction between the original metal ions and the organic ligand. Tsuruoka et al. prepared 3D MOF and finally obtained MOF nanorods with high aspect ratios by adjusting the addition amount of acetic acid.^[^ ^63^ ^]^ Through characterization, it was found that the length of 391–210 nm, width of 82–23 nm, different length, and thickness of the rod nanocrystals were prepared. This experiment confirmed that acetic acid, as a regulator, could inhibit the binding of metal ions to organic ligands and limit the growth of MOF, resulting in nanorods. In the controlled mediation method, the structure and size of MOF can be controlled not only by adjusting the acetic acid, but also by adjusting the content of solvent and water involved in the reaction. Sui et al. synthesized Zn‐MOF‐47 by solvothermal method, in which nano/microscale rods with different diameters and lengths were obtained by regulating the content of water in the reaction solvent (**Figure** **3**a).^[^ ^64^ ^]^ With the increase in water content, the size of synthetic MOF also changes, and the diameter of MOF can be accurately regulated. As shown from scanning electron microscopy (SEM) characterization (Figure 3b–d), the diameter can be reduced from micrometers to tens of nanometers, and the length can also be reduced from micrometers to hundreds of nanometers. Not only does water content have an effect on the kinetics of crystal river formation, but also some linking acids. For instance, by changing the concentration of ligands, the grain size can be effectively controlled to obtain nanosized materials. The obtained nano‐MOFs, with smaller size and a clearer internal layered structure, can effectively remove organic pollution under the synergistic effect of adsorption and photodegradation.

Using controlled mediation method can not only effectively control the size of the crystal but also can affect the agglomeration state of the crystal to further affect the obtained morphology. Zhang et al. synthesized two kinds of nanosized Co‐MOFs (nano*‐*Co‐MOF) with different morphologies with or without the addition of polyvinylpyrrolidone (PVP).^[^ ^67^ ^]^ Without the addition of surface stabilizer, nano*‐*Co‐MOF sheets were deposited on the surface of nickel foam. After the addition of the surface stabilizer, due to the unique characteristics of PVP for water, the original aggregation of nanosheets gradually dispersed and finally formed a unique nanosheet array structure. Because of the participation of the regulator, two different forms of nano/microscale MOFs were prepared under the same conditions. According to the tests, both nano*‐*Co‐MOF showed good electrochemical performance, but the nanosheet array showed slightly better electrochemical activity than the microspheres clustered on the surface. Zheng et al. use the concentration of organic ligand pyridine as regulator to influence the growth dynamics of the MOF crystal and control the growth direction of MOF crystal, thus synthesizing the layered mechanism with a 3D column as the support body.^[^ ^65^ ^]^ Among them, different shapes of nano/microscale MOF crystals will be formed due to different concentrations of regulators, as shown in Figure 3e, showing 1D nanofibers, 2D nanosheets, and 3D petal‐like shapes (Figure 3f–h). The 2D nanocrystals prepared by this method can stably exist in water without any morphologic changes, so they have the potential of application as supercapacitors. Due to the shorter ionic diffusion distance and the special 3D columnar‐supported lamellar structure, the nanosheets have better specific capacitance. Supercapacitors with excellent cycling performance were obtained by connecting two water‐based asymmetric supercapacitors in series, maintaining 95% at 5000 cycles at 3 m cm^−2^ and showing a specific capacity of 612 F g^−1^ at 0.5 A g^−1^. By adjusting the concentration of the two metals, Wang et al. successfully synthesized MOFs with different morphologies and sizes.^[^ ^66^ ^]^ From Figure 3i, it can be observed that the different shapes of Ni_1−*x*_Co_*x*_ will change greatly with the increase in nickel concentration. Due to the change in the coordination force between metal ions and organic ligands, the size of nano/microscale MOFs will increase and the structure will be very different. It is obvious that some of them are coated on the surface‐like nanoparticles, and some are similar to some nanorods embedded on the surface of the sphere. In summary, the controlled mediation method is a convenient and efficient method for preparing nano/microscale MOFs, which can effectively adjust the size and morphology of nanomaterials.

### 2.2 Template Method

In recent years, researchers have done a lot of research in controlling the shape, size, and spatial structure of the prepared materials. Template method has attracted official attention because it can easily prepare nanomaterials with unique size, structure, and shape.^[^ ^68^, ^69^, ^70^, ^71^ ^]^ What's more, the synthesis of nanomaterials via the template method can be divided into three steps: the first step is to make the template, the second step is to synthesize the material on the template, and the last step is to separate the template (not necessarily). In these years, most nano/microscale MOFs present various morphologies, including flakes, flowers, and small pieces; however, some 2D nanorods with controllable length and diameter become a difficult problem for research. It is found that this difficulty can be effectively solved by the template method. This method can be synthesized using some templates with pore structure. Therefore, the template method is widely used to synthesize MOFs with stable properties that can be effectively grown on the template, such as nanorods and nanowires.

**Figure 4:**
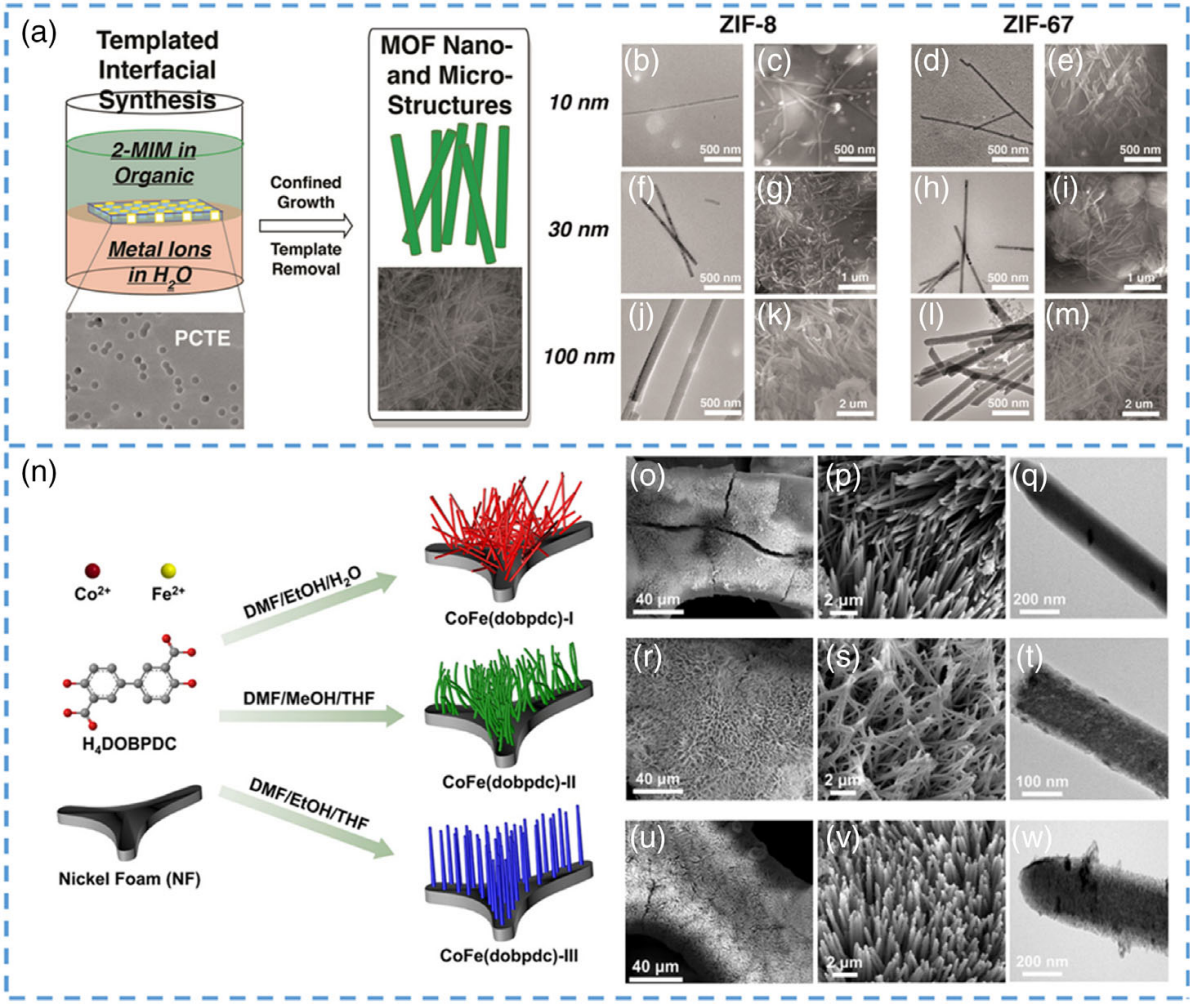
a) Schematic illustration of the synthesis of ZIF‐8 and ZIF‐67 1D nano‐ and microstructures. b–m) SEM analyses and SEM images of both organic and aqueous‐facing surfaces of track‐etched PCTE templates of various pore sizes containing ZIF‐8 and ZIF‐67 nano‐ and microstructures after interfacial synthesis. n) Schematic illustration of the synthesis of CoFe(dobpdc)‐I to CoFe(dobpdc)‐III nanorods. o–w) SEM and TEM image of CoFe(dobpdc)‐I (o–q), CoFe(dobpdc)‐II (r–t), and CoFe(dobpdc)‐III (u–w) nanorods. a–m) Reproduced with permission.^[^ ^72^ ^]^ Copyright 2021, Nature. (n–w) Reproduced with permission.^[^ ^73^ ^]^ Copyright 2021, American Chemical Society.

MOF nanostructures can be efficiently generated using pore structures in polymer or anodized alumina templates. ZIF‐8 synthesized by metal Zn^2+^ and ligand (2‐methylimidazole) has excellent chemical and thermal stability, which makes it widely used in the energy field. Meng et al., using polycarbonate (PCTE) membrane as template, and ZIF‐8 and ZIF‐67 with different sizes of 10 nm–20 μm were accurately prepared and synthesized at the oil–water interface (**Figure** **4**a).^[^ ^72^ ^]^ The researchers used films of different pore sizes and thicknesses as templates to obtain 1D nano/microscale MOF structures. The team gently placed a preprepared aqueous solution on top of the PCTE and allowed it to float on the surface of the aqueous solution. The nano/microscale MOFs were synthesized at the phase interface. After a period of time, they separated the template in chloroform to get a better look at the structure of the sample. After separation, pure nano/microscale MOFs were obtained (as shown in Figure 4b–m). The team successfully prepared nanowires and nanorod‐like ZIF‐8 and ZIF‐67 with diameters similar to the template aperture. Nanorods prepared by the above template method grow parallel and oriented on the surface of the template. The experimental conditions were adjusted and optimized in detail, and it was concluded that the size of synthesizing MOF could be effectively limited by changing the concentration of reaction solution and the aperture and thickness of the template.

Xu et al. synthesized nanorods perpendicular to the template using 3D nickel foam as a template (Figure 4n).^[^ ^73^ ^]^ The team dissolved 4,4'‐dihydroxy biphenyl‐3,3'‐dicarboxylic acid in NF mixed with a divalent metal salt of Co^2+^ and Fe^2+^ and then heated it to 120 °C. In this way, nanorods of three different shapes can be generated on the surface of nickel foam: 1) disordered and intersecting, 2) perpendicular to the surface of the formwork but also intersecting, and 3) neatly arranged perpendicular to the surface of the template. Compared with other templates such as graphene and carbon, nickel foam templates have a lower cost, high specific surface area, and flexibility. Compared with the SEM and transmission electron microscopy (TEM) characterization methods (Figure 4o–w), the results confirmed the successful preparation of MOF nanorods, consistent with the above classification results. After characterization, it was found that the prepared MOF nanorods have the high surface area and large pore channels. It is very convenient to react with electrolytes during catalysis, resulting in high catalytic activity. At a current density of 10 mA cm^−2^, it has a low potential of 176 mV and exhibits excellent oxygen evolution reactivity. The synthesis process of nanorods by this method is simple and profitable. No adhesive is used in the preparation process, effectively avoiding the resistance caused by adhesive. Second, the orderly arrangement makes the active site more thoroughly exposed, which is conducive to improving its catalytic performance. Yan et al. used nickel foam as a template, and FeCo‐MOF with nanoflower structure was grown in situ on its surface, which essentially assisted the generation of this unique nanostructure using the template.^[^ ^74^ ^]^ More importantly, the nanonetwork can be used as a template for boron‐doped graphene quantum dots (BGQDs) through the powerful interface of M—O—C bonds, avoiding π–π rearrangement and providing efficient charge transfer and abundant edge active sites. The catalyst exhibited excellent ORR performance, with its stability 58.6% higher than Pt/C catalyst and its maximum power density 1.53 times that of Pt/C electrode. The composite catalyst has enhanced electrode/electrolyte transport interface, abundant catalytic sites, and low charge transfer resistance. Recently, Chang et al. used nickel foam as a template to prepare Ni‐MOF by in situ growth.^[^ ^75^ ^]^ Through characterization, it was found that the nano/microscale MOFs sheet structures prepared on the surface were neatly arranged on the surface of the nickel foam, forming an accordion‐like structure, and there were gaps between the nanosheets. The researchers also used it in supercapacitors, an energy storage device, and subsequent electrochemical tests did not disappoint. The unique 2D nanosheet morphology provides sufficient space for ion transport and diffusion, reducing the original resistance and improving its conductivity and magnification performance. Using the template method, the researchers quickly obtained many unique nanostructures, which can be ordered or disordered to grow and arrange in irregular patterns on the template. The resulting nanostructures allow researchers to apply them to fields as diverse as catalysis, energy storage, and sensing.

### 2.3 One‐Pot Method

Currently, the one‐pot method is the most effective and widely accepted method for preparing nano/microscale MOFs, which has aroused extensive research interest.^[^ ^76^, ^77^, ^78^ ^]^ One‐pot method refers to a convenient and efficient type of organic reaction in which the reaction material is added to the reaction vessel before the response and can be performed continuously in multiple steps without other operations. The one‐pot method simplifies the process for multicomponent reactions that combine three or more components. Besides, intermediates do not need to be separated in the one‐pot method, and the more complex products’ final structure can be obtained directly in one step. Therefore, based on the above unique advantages, the one‐pot method is widely used in the preparation of multicomponent MOFs and has also become the preferred method for the preparation of micro/nanocrystals.

**Figure 5:**
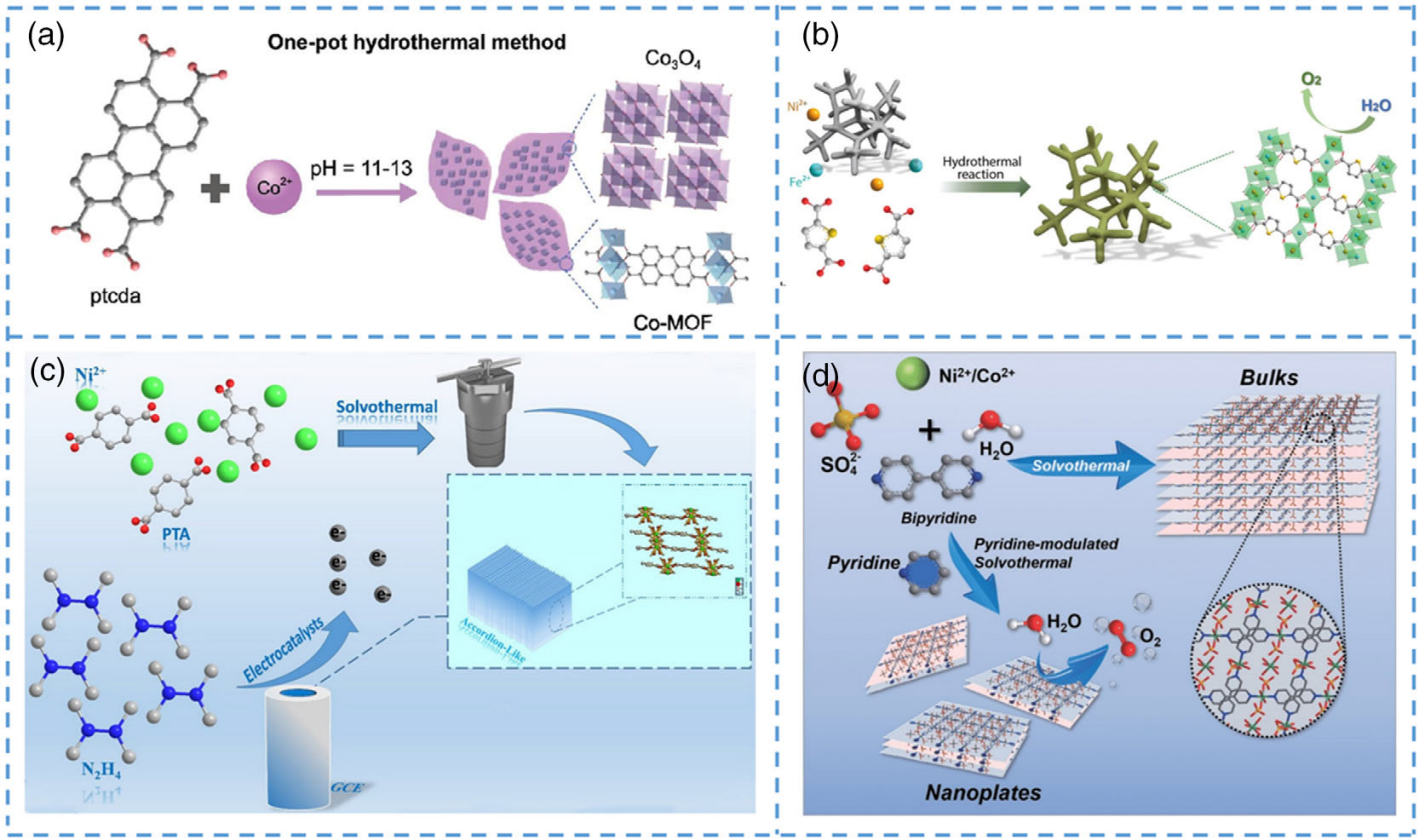
a) Co_3_O_4_@Co‐MOF schematic diagram of preparation of composite materials. b) Preparation and synthesis roadmap of FeNi‐MOF. c) Schematic diagram of preparation of Ni‐MOF ultrathin nanosheets and schematic diagram of catalytic N_2_H_4_. d) Schematic diagram of Ni/Co‐MOF nanoplates and schematic diagram of OER catalytic mechanism. a) Reproduced with permission.^[^ ^41^ ^]^ Copyright 2019, Published by Oxford University Press on behalf of China Science Publishing & Media Ltd. b) Reproduced with permission.^[^ ^79^ ^]^ Copyright 2021, American Chemical Society. c) Reproduced with permission.^[^ ^82^ ^]^ Copyright 2020, Springer‐Verlag GmbH Austria, part of Springer Nature. d) Reproduced with permission.^[^ ^83^ ^]^ Copyright 2021, Science China Press and Springer‐Verlag GmbH Germany, part of Springer Nature.

Zheng et al. designed a strategy for synthesizing nano‐MOF composites by combining metal oxides with MOF phases with a large specific surface area, leaf‐like Co‐MOF sheets were successfully prepared in a high‐alkaline environment, and nanoscale metal oxides were grown on the surface (**Figure** **5**a).^[^ ^41^ ^]^ It was found that C, O, and Co elements were dispersed on the surface of the foliated MOF. The composite MOF crystal structure simultaneously combines the advantages of both metal oxide and MOF to maximize the edges, not only having the unique benefits of each component but also contributing to the strength of the whole material. MOF has a large surface area and pore structure, which enables the material to provide many channels for electrochemical reaction and cation movement when applied to the energy storage process. Moreover, the existence of metal oxides provides more active sites based on the original metal sites. The test shows that the composite can maintain original morphology and characteristics in alkaline solution and offer relatively good stability. In the subsequent electrochemical performance test, it is made into the electrode material of the supercapacitor. The specific capacitance of the supercapacitor can reach 1020 F g^−1^ in 3.0 m KOH. In addition to high specific capacitance, it also has good stability. Even at a high current density, the capacitance retention rate can be maintained above 96.7%. In addition to the superposition of metal oxides on the surface of MOFs, pure MOFs can be effectively prepared by the one‐pot method and are limited to not only MOFs of single metal elements but also MOFs of multiple metal components that can be synthesized efficiently. In a recent study, Wang et al. successfully prepared FeNi‐MOFs with efficient oxygen evolution reaction (OER) catalytic properties by a one‐pot method and exhibited a nanoarray‐like arrangement of shapes.^[^ ^79^ ^]^ The researchers succeeded in preparing FeNi‐MOFs (shown in Figure 5b) after a reaction at 180 °C for up to 22 h, and after SEM characterization, they were found to be rod shaped, with a thickness of around 79 nm. In addition, they also tried to prepare Fe and Ni monometallic MOFs using a unified one‐pot method. They confirmed that they have a single‐crystal structure by SEM and other characterization methods, which indicate that both mono‐ and multimetallic elemental MOFs are dependent on the one‐pot method. Wang et al. selected Ni or Cu as weak electrophilic metals and strong oxidizing aromatic linkers to synthesize a series of conductive MOFs with robust π‐conjugated M–L covalent centers by the one‐pot method.^[^ ^80^ ^]^ These studies mentioned above further confirmed that one‐pot method could be used to prepare a list of nano/microscale MOFs with superior single crystallinity and phase purity and have universality. In addition, in Chen's study, NiCo‐MOF‐74 was also synthesized, which has good electrical conductivity as a cathode material of nickel–zinc batteries.^[^ ^81^ ^]^

In addition to the aggregated sheet structure, the one‐pot method can also directly prepare ultrathin nanosheets. The 2D nanosheet structure, as a fundamental important classification among many branches of the MOF structure, has attracted much attention from researchers. The ultrathin nanosheet structure is prepared by nanocrystallization of large particles of MOF. The nanostructure can expose the active sites contained in the structure to the maximum extent, significantly improve the material's conductivity, and has great application potential in the catalytic field. In the application of energy storage devices, the catalytic performance of MOFs can be brought into full play when the structure is nanoflakes. Therefore, most electrode materials will be prepared by MOFs with nanoflake morphology. Thus, nowadays, many researchers have transformed the original structure of MOFs into ultrathin 2D MOF nanosheets to improve its catalytic performance. However, with the increase in catalytic activity, the stability of the material will be reduced. Interestingly, some unique structures can effectively avoid this situation. Cao et al. prepared ultrathin Ni‐MOF nanosheet crystals by the one‐pot method, demonstrating high catalytic performance and sensitivity to N_2_H_4_ (Figure 5c).^[^ ^82^ ^]^ The nanosheet presented an accordion‐like arrangement. This unique arrangement dramatically improves the stability of the material and makes the inorganic material have good strength while maintaining the high activity of the nanomaterial. It is a feasible method for constructing hierarchical nanostructures. Therefore, accordion‐like nanostructures are recognized as the most suitable nanostructures for catalytic applications. As a result, the team synthesized an accordion‐like Ni‐MOF assembled from ultrathin nanosheets, which performed well in catalytic tests and had potential applications for N_2_H_4_ sensing. In addition to the accordion‐like ultrathin nanosheets, some other 2D nano/microscale MOFs structures also have good catalytic performance. Bai et al. prepared bimetal‐doped MOF nanoplates with a thickness of about 20 nm through a simple one‐pot method and nanoplates with an aspect ratio greater than 50 (Figure 5d).^[^ ^83^ ^]^ One‐pot method is used, and pyridine is used as a regulator to intervene and regulate the growth dynamics of MOF crystals. The mediation control method and the one‐pot method are effectively combined to prepare controllable nano/microscale MOF crystals. It was found that the prepared nanoplates had prominent nanoappearance, uniform size, and smooth surface. Pyridine solution was used as a regulator to limit the growth of MOFs in the vertical direction, thus achieving the purpose of preparing nanocrystals. The coordination of organic ligands and metal atoms inhibits the aggregation of nanoplates and avoids the accumulation of layers, thus forming a 2D layered structure. By adjusting the concentration of the pyridine solution, the team made microscale MOF crystals in addition to the nanoscale MOF. In the subsequent test of electrocatalytic performance, the catalytic performance and long‐term cyclic stability were demonstrated. It can be seen that the one‐pot method is a very efficient and convenient method for the synthesis of nanoscale MOF.

### 2.4 Top‐down Method

With the intensive research of nanotechnology, more researchers are focusing on applying the top‐down method in nanomaterial preparation, which is usually more efficient and environmentally friendly than other methods. MOF structures usually contain some chemical bonds, strong bonding layers, and weak bonding layers. Using the top‐down method, the material properties can be optimized by dividing the cylinder or block into several small pieces or aggregating the small pieces into large pieces. Usually, the MOF is a large or columnar structure with multiple layers, and some physical interventions can successfully exfoliate the multilayer MOF. For weak interactions between adjacent layers, the original 3D structure can be transformed into a 2D structure by exfoliation through ultrasound, vibration, and mechanics.^[^ ^84^, ^85^, ^86^ ^]^ Especially in the preparation of MOF nanosheets, the top‐down MOF nanosheet preparation method is less limited and more applicable compared with other methods.

**Figure 6:**
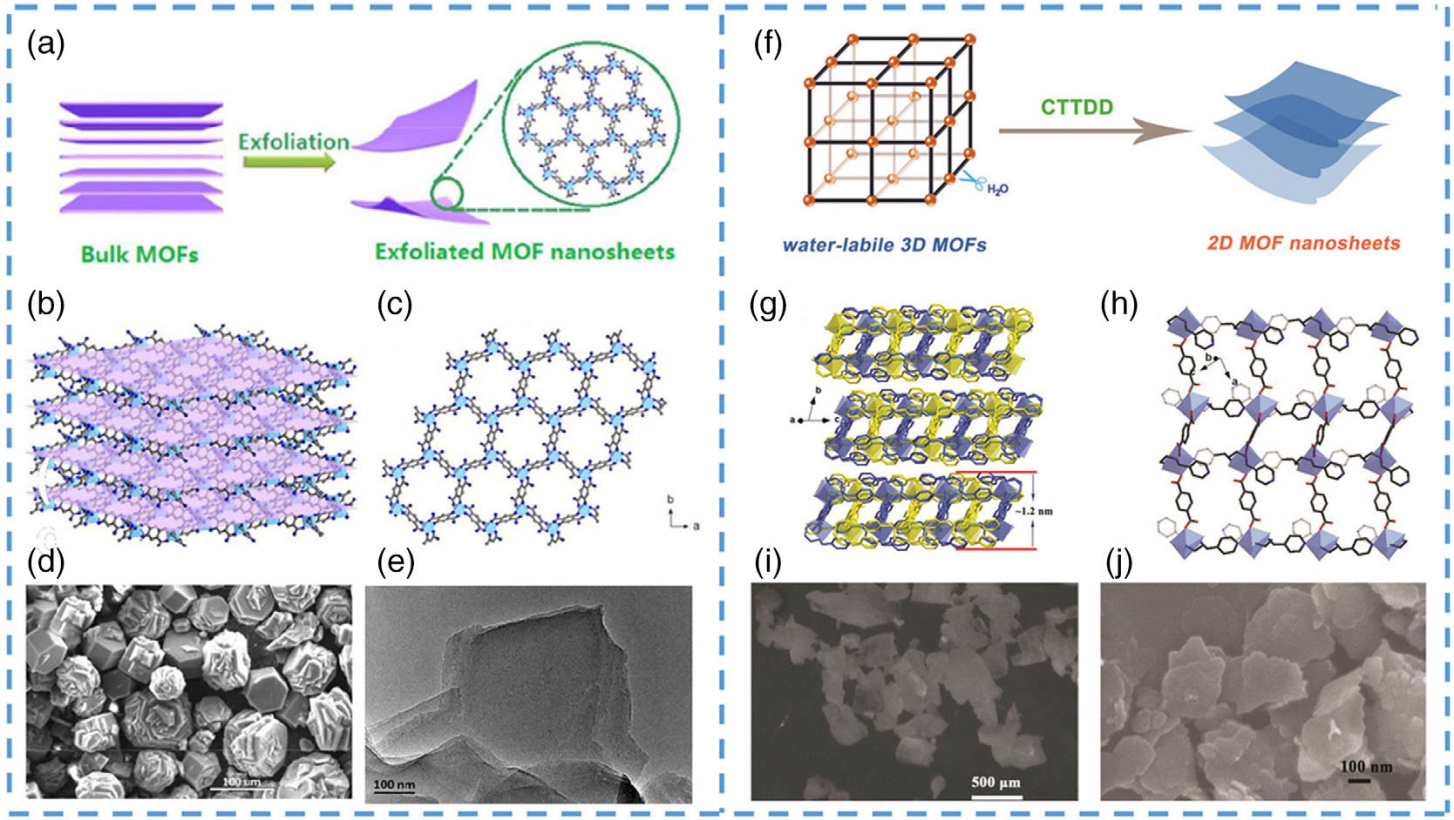
a) The schematic illustration for the fabrication of the 2D MOF nanosheets materials. b) Architecture of the layered MOF precursor. c) Illustration of the honeycomb‐like layered structure of NTU‐9‐NS. d) SEM image of as‐synthesized NTU‐9 crystals. e) TEM images of NTU‐9‐NS morphologies. f) The schematic illustrations of the CTTDD strategy to fabricate 2D MOF nanosheets. g) 3D supramolecular structure of HSB‐W5 and the thickness of a single layer. h) 2D layer of HSB‐W5 viewed along the *b*‐axis. i–j) SEM images of the samples: (i) pristine HSB‐W1, (j) HSB‐W5‐Ns. a–e) Reproduced with permission.^[^ ^88^ ^]^ Copyright 2013, Royal Society of Chemistry. f–j) Reproduced with permission.^[^ ^91^ ^]^ Copyright 2020, Royal Society of Chemistry.

In recent years, many successful cases have separated multilayer MOFs to form individual ultrathin MOF nanosheets.^[^ ^84^, ^85^, ^86^, ^87^ ^]^ Xu et al. obtained ultrathin nanocrystal MOF material from top to bottom by a self‐assembly method.^[^ ^88^ ^]^ After testing, it was found that the nanocrystal could respond quickly when faced with ferric metal ions, so it has excellent application prospects in sensing (**Figure** **6**a). The team used a convenient method to prepare the precursor NTU‐9, characterized by a hexagonal prism crystal shape (Figure 6d). The crystal is composed of multiple layers connected by hydrogen bonds between layers and the coordination bonds formed by Ti^4+^ and oxygen atoms in the organic ligand within the layers, showing regular honeycomb and hexagonal channels (Figure 6b,c). This multilayer structure provides the opportunity to strip into a single layer of nanosheets. After a long time of ultrasonic treatment in isopropanol solution at room temperature, it was used to destroy the hydrogen bonds between layers. Through characterization, it was observed that the morphology changed from block to flake obviously, and the transverse diameter was about 100 nm or smaller (Figure 6e). The prepared nanosheets have high sensitivity to Fe^3+^ and can respond to the ion quickly in a short time with high sensitivity. Response times can be maintained in seconds, making it a standout among many MOF materials. From large crystals to ultrathin nanosheets, it is important to deal with the bonds or interactions that connect the layers, but the smaller the forces that bind the layers, the better the self‐assembly of nanostructures. Multiple hydrogen bonds make it difficult to separate the multilayer structure. The separation would be easier if the interaction force is not as strong as the hydrogen bonds. With this idea in mind, MOFs that cannot exist stably in the water came into the view of researchers. Such MOFs are usually insoluble with water, and the original structure will collapse or undergo phase transformation with the change of organic ligand or hydrolysis of the network. Therefore, when the 3D MOF with unique physical properties is placed in an aqueous solution, it can spontaneously assemble into a laminated nanostructure, and the interaction force between the connecting layers is destroyed.^[^ ^89^, ^90^ ^]^ Therefore, the self‐assembly method has great application potential in preparing ultrathin MOF nanosheets, and many researchers have made attempts. Wen et al. obtained ultrathin nanosheets by the characteristics of unstable MOFs in water through self‐assembly (Figure 6f).^[^ ^91^ ^]^ The team prepared a columnar precursor of MOF with a simple approach, consisting of multiple layers inside (Figure 6g,h). Then it is placed in an aqueous solution. With the participation of water molecules, the coordination bonds between the metal and the organic ligand are destroyed, thus forming unstable coordination bonds between metal ions and water molecules. This process completes the transformation from the original block column to a sheet. Subsequent characterization also confirmed the formation of double‐layered nanosheets (Figure 6i,j). The two layers are connected to form a more stable double‐layer interspersed structure. The subsequent thinner nanosheets provided an excellent precursor. Through ultrasonic intervention, monolayer nanosheets with the transverse size of about 300 nm were successfully obtained, which have excellent potential for application in the sensing field. The main principle of this method is to destroy the interaction force of the connection in the layered structure and break the initially stable coordination bond with the participation of some molecules. It is a green, convenient, and effective method for preparing MOF nanosheets.

### 2.5 Interfacial Growth Method

**Figure 7:**
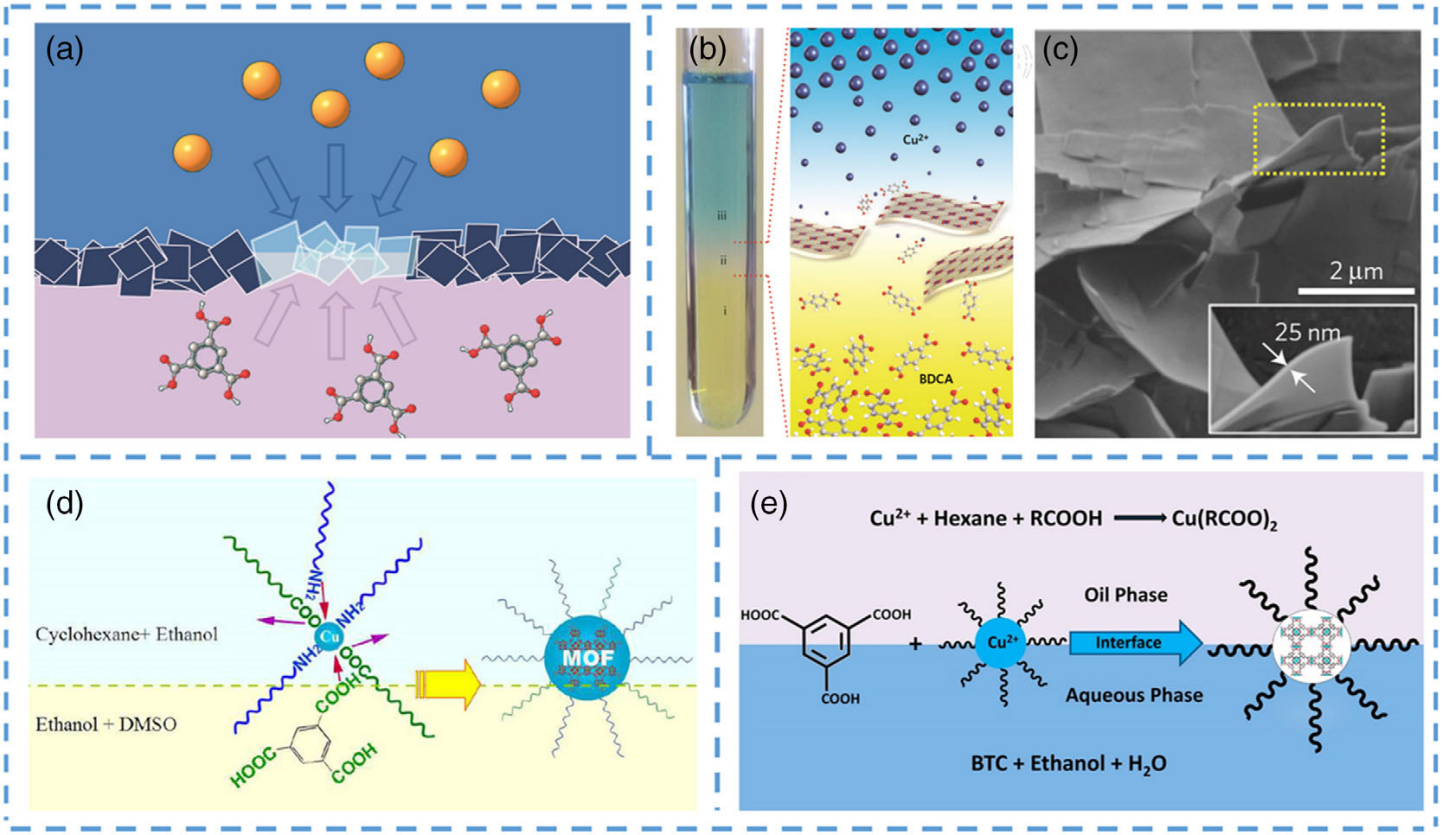
a) Schematic diagram of preparing nanometer MOF at interface of two‐phase mixture. b) Picture showing the spatial arrangement of different liquid layers during the synthesis of CuBDC MOF nanosheets. c) SEM image for CuBDC MOF nanosheets synthesized. d) Scheme of the proposed MOF nanocrystal formation mechanism. e) Formation mechanism of OA‐capped nanosized HKUST‐1 prepared by the LSS method. a) Reproduced with permission.^[^ ^93^ ^]^ Copyright 2011, Springer Nature. b,c) Reproduced with permission.^[^ ^87^ ^]^ Copyright 2015, Springer Nature. d) Reproduced with permission.^[^ ^95^ ^]^ Copyright 2016, Royal Society of Chemistry. e) Reproduced with permission.^[^ ^96^ ^]^ Copyright 2019, American Chemical Society.

Interface, as a necessary way of ion transport, plays a vital role in the dynamics of nanocrystals. Many researchers guide the formation of crystals by adding or adjusting different organic solvents, which makes the interface growth method gradually enter the field of vision of researchers.^[^ ^92^ ^]^ The interfacial growth method usually refers to a two‐phase or liquid–liquid interface, where metal ions are provided on one side of the interface, and organic ligands are provided on the other side. Finally, they contact each other on the interface and generate nano/microscale MOFs (**Figure** **7**a).^[^ ^93^, ^94^ ^]^ Its synthesis is similar to the template method because it uses an interface to grow the material. However, the template in the template method is not involved in the reaction, so the template needs to be removed after the material is generated. The interfacial growth method avoids this problem by fully using the phase interface. This method can control the thickness and shape of MOF crystals synthesized at the interface by adjusting the concentration of precursor and the type of interface and is usually used to synthesize flake, shell, and small‐particle MOF crystals.

Rodenas et al. mixed copper ions, *N*,*N*‐dimethylformamide (DMF), and cosolvent and obtained nanosheets with a thickness of about 25 nm at the interface (Figure 7c).^[^ ^87^ ^]^ They took advantage of the difference in density between the three solutions to cause the mixture to stratify spontaneously (Figure 7b). The obtained nanosheets displayed a square layer morphology with a thickness of 5–25 nm and lateral dimensions of 0.5–4 mm. The team also managed to control the thickness of the synthesized nanosheets by changing the reaction temperature. The interfacial growth method can produce not only nanosheets but also nanoparticles. Xu et al. constructed a dual‐solvent system using two ligand solvents and synthesized MOF nanoparticles at the liquid–liquid interface.^[^ ^95^ ^]^ Alkyl amines and alkyl carboxylic acids are used as ligand‐providing solvents on copper ions (Figure 7d). Compared with the previous synthesis method, the speed of the two solvents for the ligand of MOF nanoparticles is different, so that the two ligand solvents cooperate. It greatly promoted the nucleation of small‐sized nano/microscale‐MOFs, inhibited the primitive disorderly growth, and controlled the diameter of newly generated MOF particles within 10 nm. The interfacial growth method not only occurs at liquid–liquid interface but also applies to the liquid–solid–liquid interface. Cai et al. synthesized 30–140 nm MOF crystals at the water–oil interface using interfacial growth method (Figure 7e).^[^ ^96^ ^]^ At the interface between ethanol‐n‐hexane liquid and water–ethanol solution, owing to the generation of copper(II) oleate clusters and the protection of OA, it results in the the formation of highly uniform nanoparticles.

### 2.6 Microwave/Ultrasonic Radiation Method

The template, interface growth, and other traditional methods have been introduced before, but when the reaction requires a lot of heat and energy, these traditional methods are difficult to meet the requirements, and the control of conditions is also difficult. With the help of the microwave, this problem can be solved, and nanomaterials can be prepared quickly under mild conditions. This method is faster, greener, and safer than traditional methods and has been widely used in the preparation of materials in recent years. Microwave radiation can make the solution quickly reach a higher temperature, to a certain extent to avoid the generation of excess crystals (such as precursors). Metal ions can selectively absorb the energy provided by microwaves and provide a large number of reaction sites, which significantly improve the finger front factor of the Arrhenius equation for crystal nucleation. It can also make the solution obtain a large amount of energy, which directly affects the crystal nuclear dynamics and is generally applicable to the synthesis of MOFs that requires high reaction conditions, high temperature, or high energy.^[^ ^97^, ^98^ ^]^ Due to the high requirements of researchers on crystal size, the continuous improvement of synthesis methods, and the high selectivity and efficiency of microwave radiation method, this method is widely used in the study of MOF size control.

**Figure 8:**
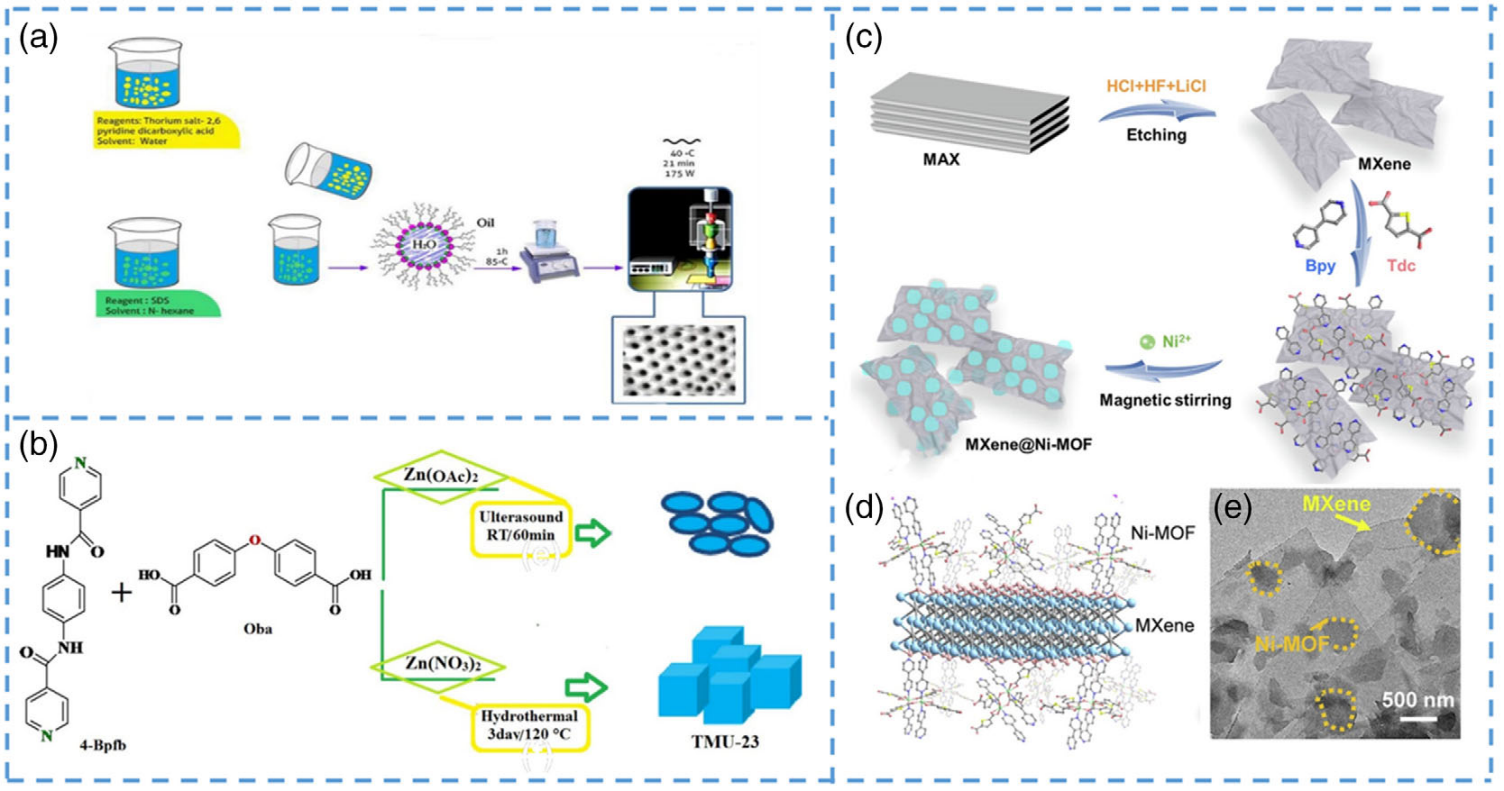
a) Schematic representation of Th‐MOF sample synthesized under optimal conditions of UARM method. b) Material produced and synthesis method. c) Schematic illustration of the preparation of MXene@Ni‐MOF. d) Diagram of the structure of the MXene@Ni‐MOF. e) The MXene@Ni‐MOF of TEM image. a) Reproduced with permission.^[^ ^100^ ^]^ Copyright 2017, Elsevier. b) Reproduced with permission.^[^ ^101^ ^]^ Copyright 2018, Elsevier. c–e) Reproduced with permission.^[^ ^65^ ^]^ Copyright 2022, Elsevier.

In most of the ultrasonic nanocrystals, regulators are added in the synthesis process. When the regulator is not added, the obtained MOF crystal size will be too large. Bunzen's group did some research.^[^ ^99^ ^]^ They modified the solution in the microwave environment by adding a base solution as a modifier and found that the size of the synthesized crystal was reduced to 100 nm. This study lays a foundation for the subsequent application of microwave synthesis. Sargazi et al. successfully prepared nano‐TH‐MOF by an ultrasonic method.^[^ ^100^ ^]^ Aqueous solutions of 2,6‐pyridine dicarboxylic acid and thorium nitrate (IV) pentahydrate were prepared as the reaction solution, and then sodium dodecyl sulfate and n‐hexane solutions were added, fully stirred, and placed in an ultrasonic bath. After ultrasonic irradiation for 21 min, nano‐TH‐MOF nanocrystals were finally formed (**Figure** **8**a). By varying the sonication time and the sonication power, nanosized Th‐MOF with different morphologies were obtained, and these crystals varied greatly in size and morphology, some being flaky and others smooth and spherical. Several studies have shown that the involvement of ultrasound greatly affects the size and shape of the crystal. Gharib et al. synthesized TMU‐23 with nanometer size by ultrasonic irradiation of the precursor at room temperature.^[^ ^101^ ^]^ They placed the dehydrated zinc acetate solution in a high‐power ultrasonic probe and added two ligands at normal pressure and temperature for 1 h (Figure 8b). In order to find the best morphology, the team also adjusted the ratio of DMF, and by comparison, the best crystallization was achieved when the DMF concentration was 0.03. In addition, the ultrasonic time also has an impact on the morphology of the crystal, and too long time will lead to crystal structure fragmentation and agglomeration, resulting in poor performance as a solid sensor.

Moreover, water bath ultrasound is also a key step in the preparation of nano‐MOF. Zheng et al. synthesized and prepared Ni‐MOF nanosheets, in which the key step is to compound with MXene in the water bath in an ultrasonic instrument to obtain stable 3D materials as MXene@Ni‐MOFs (Figure 8c).^[^ ^65^ ^]^ In this material, the interaction between the surface functional groups of MXene itself and the Ni‐MOF organic ligand delays the service life of MXene and greatly increases its spacing, which greatly optimizes the original conductivity (structure diagram is shown in Figure 8d,e). The synergistic effect of the two can not only improve the conductivity, but also effectively prevent the agglomeration between the nanosheets, so that the flake structure can be maintained. The special columnar 3D structure, compared with the single‐sheet structure, has higher structural stability, which is very conducive to the application of energy storage devices. The team prepared it for use as electrode material for supercapacitors and tested its composite 3D material for high specific capacity and cycle stability. However, remarkable progress has been made for the synthesis of MOFs by a microwave‐assisted method. The defects in microwave reactors still hinder the further development of this technology. For example, microwaves can bring heat source, but they cannot completely avoid the influence of the reaction caused by local overheating. Zhao et al. explored the simulation of the synthesis of MIL‐88B (Fe) under different microwave reaction conditions.^[^ ^102^ ^]^ Based on the results of numerical simulations, several optimization strategies for microwave‐assisted synthesis of MOFs were proposed to make the reactants heated uniformly and avoid concentrated overheating, bringing about a more uniform size distribution and more regular shape of the synthesized nano/microscale MOFs. This provides a new idea for the application of ultrasonic method in the preparation of nano/microscale MOFs and expands the application potential of ultrasonic method in the preparation of nanocrystals.

### 2.7 Summary

In general, hydrothermal/solvothermal methods are the most common one‐pot methods for the synthesize synthesis of nano/microscale MOFs with controllable structures (nanoparticles, nanorods, and nanoflowers), which are simple and have wide applicability. The one‐pot synthetic method depends on the rate of reduction of metal ions and/or the growth rate of MOFs. The controlled mediation method usually adopts regulators, common regulators such as acetic acid, PVP, and water. When changing the types and additional amounts of the regulators, the diameter of the synthesized nanocrystals can be controlled in the range from tens of nanometers to a few micrometers, and the length can be regulated as needed. In addition, the template method can be used to further regulate the size of nano/microscale MOFs more accurately. Some common templates, such as PCTE and NF, can be selected according to the material requirements of the appropriate template aperture. However, its disadvantages including complex preparation and high cost limit the synthesis of nano/microscale MOFs.

Interface growth method and top‐down method are effective methods to obtain 2D nanosheets structures with the thickness of <10 nm. The synthetic mechanism of the above two methods is distinct. The former uses the phase interface to combine metal ions and organic ligands on the phase interface and finally obtains the nanosheet. The latter relies on unstable bond states in MOFs itself, such as van der Waals force and hydrogen bond. By breaking the lamellar structure, the nanosheet is obtained. In addition, the interfacial growth method is also suitable to synthesize nanoparticles. The microwave radiation method can offer phase selectivity, fast crystallization, and control over the crystal morphology to synthesize nanoparticles in a short time. However, the microwave radiation method still faces some challenges in the synthesis of nano/microscale MOFs, such as local overheating and inaccurate adjustment. Overall, the designed structures for pristine MOFs are still challenging because of the weak understanding of self‐assembly, especially under invisible synthetic conditions, resulting in the changes or differences of the composition, surface area, and pore size.

## 3 Advantages

The characteristics of MOF materials can be roughly divided into the following aspects. The first is the more porous structure in the crystal structure, which is caused by the bonding between metal ions and organic ligands. In some studies today, MOF materials have not only porous structures but also multilayer structures. For electrochemical reactions that usually occur at the active sites in the material, porous and multilayer structures can ensure that the active sites are evenly distributed in the crystal structure. This feature is also very conducive to the introduction of more active sites in MOF crystals, such as adding some precious metals. The second major feature is the adjustability of the metal nodes, which is particularly important for improving electrochemical activity. For example, in the crystal of a bimetallic active site, electron transfer can be faster by adjusting the metal node so that the material has good conductivity and stability. The third feature is the selection of organic ligands. The entire frame of MOF is derived from organic ligands, and an appropriate frame can make the active sites more evenly distributed within the crystal.

Obviously, the properties of the material have a huge impact not only on the chemical properties but also on the physical properties, so that the performance of excellent application properties greatly depends on the structure and properties of the material itself. In view of the limitations of conventional MOF materials, a large number of recent studies have converted large‐ to small‐sized micro/nano‐MOFs, and surprisingly, it has been found that the shrunken materials have been greatly improved in terms of electrochemical properties. Not only in the field of electrochemical catalysis, micro/nano‐MOFs have excellent catalytic ability, but also in the field of energy storage, they have gained far‐reaching application potentials with greater capacity and cycling stability of special structure. Therefore, the following section will focus on the optimization of the electrochemical properties of nano/micro‐MOFs compared with those without shrinkage, both in terms of catalysis and in terms of energy storage, in addition to an overview of some of its advantages in other fields.

### 3.1 Enhanced Catalytic Activity for Energy Conversion

Today, the world is facing a variety of energy problems, and the original nonrenewable energy is gradually short; now it is very necessary to obtain clean, environmentally friendly renewable energy, and catalyst is the key to obtaining such energy. MOF monomers are widely used in catalysis, but there are still some disadvantages. In addition, nanoprocessing enables MOFs to obtain a larger specific surface area, expose abundant active sites in MOFs, and shorten the ion transport distance, which are very beneficial in improving its application in the field of catalysis. Next, we will summarize and compare the work of MOF nanoprocessing from these three aspects.

**Figure 9:**
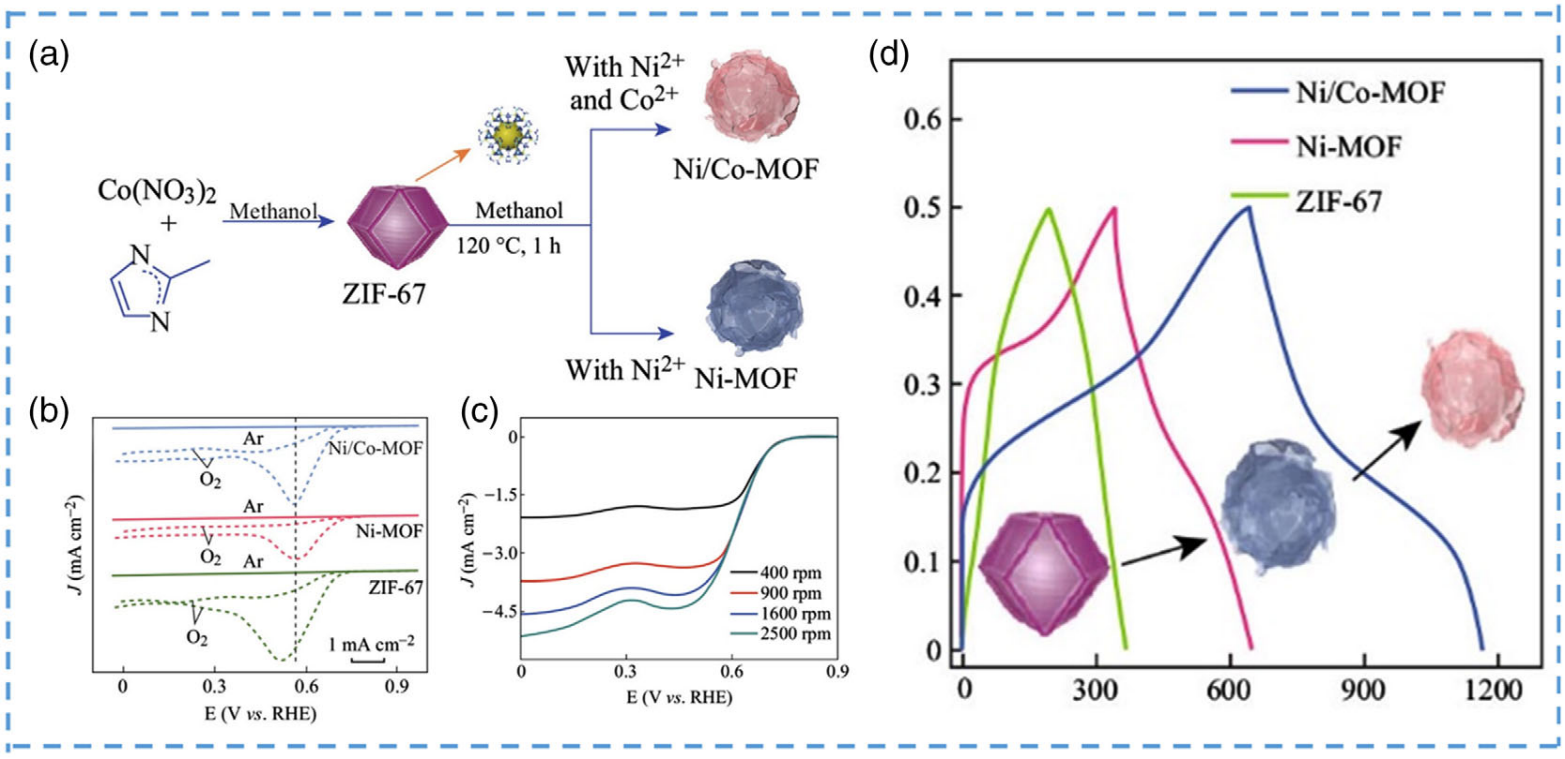
a) Schematic illustration of the synthesis of Ni/Co‐MOF nanoflakes and Ni‐MOF nanoflakes. b) CV curves in Ar‐saturated (solid curves) and O_2_‐saturated (dashed curves) solutions with a sweep rate of 50 mV s^−1^. c) Rotating disk electrode polarization curves of Ni/Co‐MOF nanoflakes at different rotation speeds. Scan rate: 10 mV s^−1^. d) Galvanostatic charge–discharge curves comparison of Ni/Co‐MOF nanoflakes, ZIF‐67, and Ni/Co‐MOF nanoflakes at various current densities in 1 m LiOH solution. a–d) Reproduced under the terms of the CC‐BY Creative Commons Attribution 4.0 International license (https://creativecommons.org/licenses/by/4.0).^[^ ^104^ ^]^ Copyright 2017, The Authors, published by Springer Nature.

Nanotechnology is the reduction of a larger size; the first result is the increase of surface area. The increase of surface area is beneficial to the optimization of catalytic effect, and many studies have confirmed this conclusion. Li et al. prepared NU‐1000 nanoparticles of different sizes and tested the hydrolysis rate of the material.^[^ ^103^ ^]^ They obtained crystals of different sizes by adjusting the concentration of benzoic acid. Among them, the largest reaches 1200 nm, and the smallest size is 75 nm. According to the experiment and calculation, with the increase in crystal size, the proportion of interposes and micropores in the internal structure also increases. The material was then observed, and the rate of catalytic hydrolysis of methyl paraoxon was used as the evaluation standard. It is obvious from the result that the hydrolysis rate decreases with the increase of particle size, and particle size has a great influence on the hydrolysis rate. The half life of methyl paraoxon hydrolyzed at room temperature is shortened as the crystal size decreases. The solution will go into the smaller nanocrystals more quickly than the medium‐sized crystals, which is also a key factor affecting the rate. To analyze the difference between the microcrystalline and nanosizes, they assumed that the crystals were ideal prisms and calculated the surface area of crystals of different sizes. By calculation, the exposure area of the microcrystalline MOF is much smaller than that of the nanometer MOF. According to the above experimental calculation, the surface area can be significantly increased after micro/nanocrystallization, and the catalytic activity is highly dependent on the surface area, which makes the catalytic activity of nano/microscale MOF significantly higher than that of the bulk MOF. To further investigate the effect of surface area on oxygen reduction catalysis, Xia's group synthesized Ni/Co‐MOF nanosheets in a simple way (**Figure** **9**a) and assembled them into microspheres to further expand their surface area.^[^ ^104^ ^]^ The microspheres are mainly composed of many 2D nanosheets. Through characterization, it is found that the nanosheets are composed of nanowires of about 1 nm, with a uniformly distributed microporous structure. In the electrochemical test, in order to compare the catalytic effect of Ni/Co‐MOF, ZIF‐67 was also tested. The nanosheet showed an obvious catalytic peak in the cyclic voltammetry (CV) diagram, and an obvious oxygen reduction peak cathode peak appeared at 0.57 V (Figure 9b). In the linear sweep voltammetry (LSV) curve, the initial potential of the nanosheet is much higher than that of the others (Figure 9c,d). It was found that the catalytic effect of MOF after nanocrystallization was significantly better. In addition, it is found that the material has good stability and selectivity through testing and has great application potential in ORR applications. The above studies and tests can prove that the surface area expanded by nanocrystallization has a positive effect on enhancing the catalytic effect of the catalyst.

**Figure 10:**
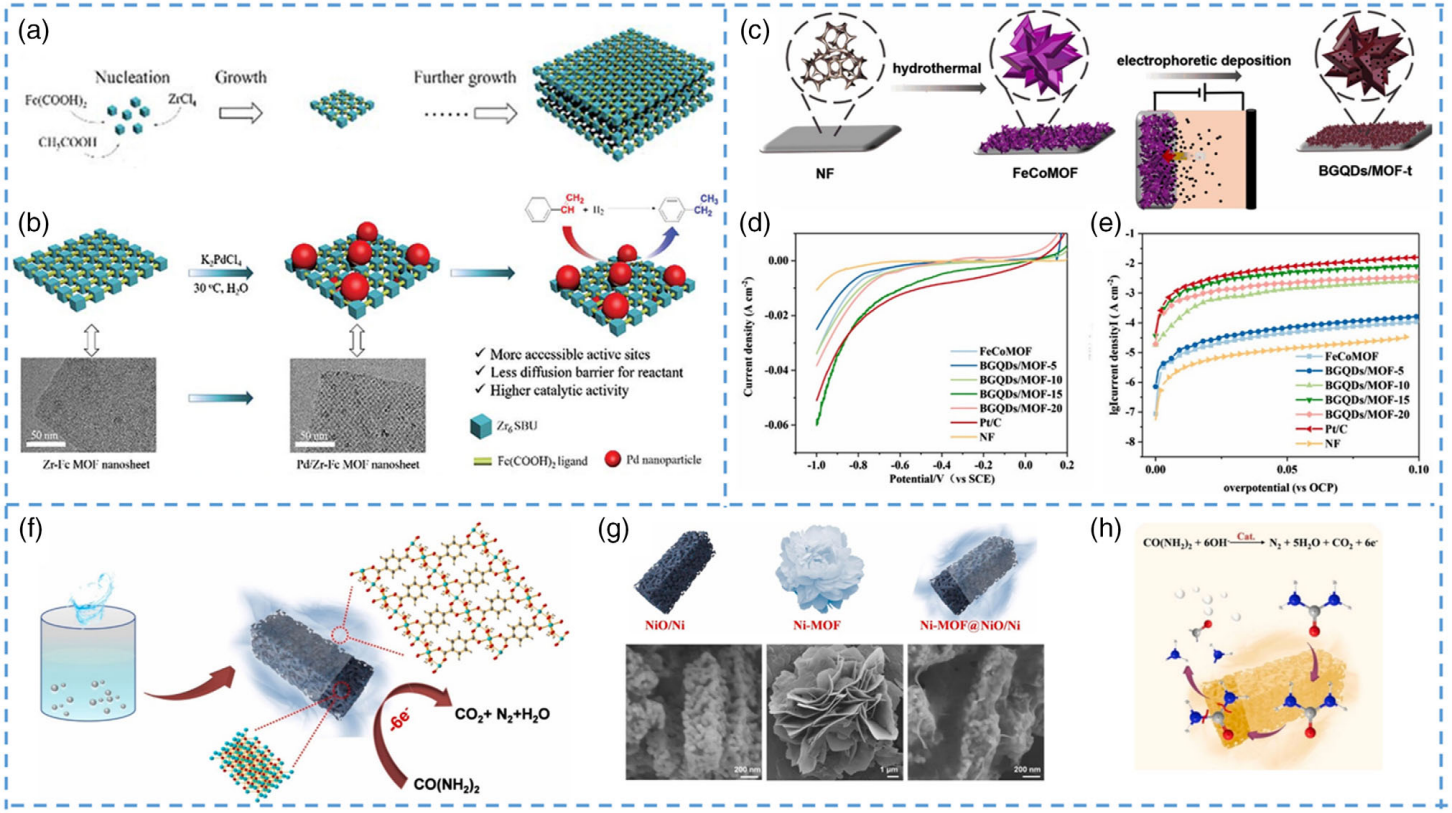
a) Proposed growth mechanism of Zr‐Fc MOF. b) Schematic of the preparation of Pd/Zr‐Fc MOF nanosheets. c) Schematic illustration of the fabrication process of the BGQDs/MOF‐t. d) LSV curves of various cathode catalysts in O_2_‐saturated PBS solution. e) Tafel plots of various cathode catalysts. f) Schematic illustration of the synthetic process of nanosheets@nanorod structure. g) Rod‐like NiO/Ni, flower‐like nanosheets Ni‐MOF, ultrathin nanosheets Ni‐MOF@NiO/Ni‐2 nanorods of schematic diagram of morphology and SEM. h) The schematic of Ni‐MOF@NiO/Ni‐2 nanorods used in UOR catalytic. a,b) Reproduced with permission.^[^ ^110^ ^]^ Copyright 2019, Royal Society of Chemistry. c–e) Reproduced with permission.^[^ ^74^ ^]^ Copyright 2022, Elsevier. f–h) Reproduced with permission.^[^ ^115^ ^]^ Copyright 2021, Elsevier.

In addition to that, it exposes the active sites that are inside. The researchers investigated the effect of nanocrystalline exposure on catalytic activity in various reactions. In the field of scientific research and in the field of industrial application, hydrogenation reaction has a pivotal position; it is widely used in many fields such as fine chemicals and pharmaceutical production.^[^ ^105^, ^106^, ^107^, ^108^ ^]^ Affected by this, the study of related catalysts becomes particularly important, such as some traditional metal oxides or sulfide catalysts. These catalysts have high catalytic activity but are easily poisoned by some elements, thus losing their original high activity.^[^ ^109^ ^]^ Soon, the potential of MOF as a catalyst was gradually discovered, and its high active site and adjustable size and morphology have attracted researchers deeply. Many experiments have been done. Deng et al. prepared MOF nanosheets using acetic acid as regulator without adding other surfactants.^[^ ^110^ ^]^ They chose bottom‐up rather than top‐down approaches to obtain flake crystals by stripping. By adjusting the concentration of acetic acid, Zr‐Fc MOF nanosheets were finally obtained (as shown in **Figure** **10**a). According to the characterization method, it was found that the nanosheet was not formed directly but was composed of nanoparticles. Due to the participation of acetic acid solution, the trend of longitudinal growth of nanoparticles was inhibited, but only lateral growth, resulting in the formation of flake structure. The nanosheets were tested and found to have a high hydrogenation catalytic capacity on styrene, in addition to which their activity did not diminish after several cycles, which is due to the structural advantages of the nanosheets (Figure 10b). Compared with other hydrogenation catalysts, this unique MOF nanosheet avoids the disadvantage of poor stability, making MOF nanosheets a potential multiphase catalyst with great application potential.^[^ ^111^, ^112^, ^113^, ^114^ ^]^ The improved catalytic effect of micro–nano MOFs due to the exposure of more active sites has been demonstrated not only in hydrogenation reactions but also in the oxygen reduction reaction (ORR), OER, and hydrogen precipitation reaction (hydrogen evolution reaction (HER)) in recent years.^[^ ^74^, ^115^, ^116^, ^117^ ^]^ Yan et al. successfully prepared FeCo‐MOF nanoflower structures (Figure 10c) by the solvothermal method.^[^ ^74^ ^]^ This morphology is very favorable for electron transport and mass transfer, so many catalysts have similar flower structures. The flower‐like MOF catalyst composed of nanocrystals was found to have higher catalytic activity than other catalysts, with enhanced electrode/electrolyte transport interface, abundant catalytic sites, and lower charge transfer resistance, exhibiting excellent ORR activity, superior to that of commercial Pt/C catalysts (Figure 10d,e). In addition to this MOF sheet, the catalytic performance of the nanorods MOF has also been improved. Li et al. developed a facile approach for preparing ultrathin nanosheet MOF@NiO/Ni nanorods (Figure 10f).^[^ ^115^ ^]^ The team successfully combined the porous carbon structure with ultrathin Ni‐MOF nanosheets through modulation (Figure 10g). The prepared composites exhibited superior electrocatalytic performance for urea oxidation reaction due to the large number of active sites exposed by their hierarchical nanosheet–nanorod structures, abundant Ni species, and improved stability (Figure 10h).

**Figure 11:**
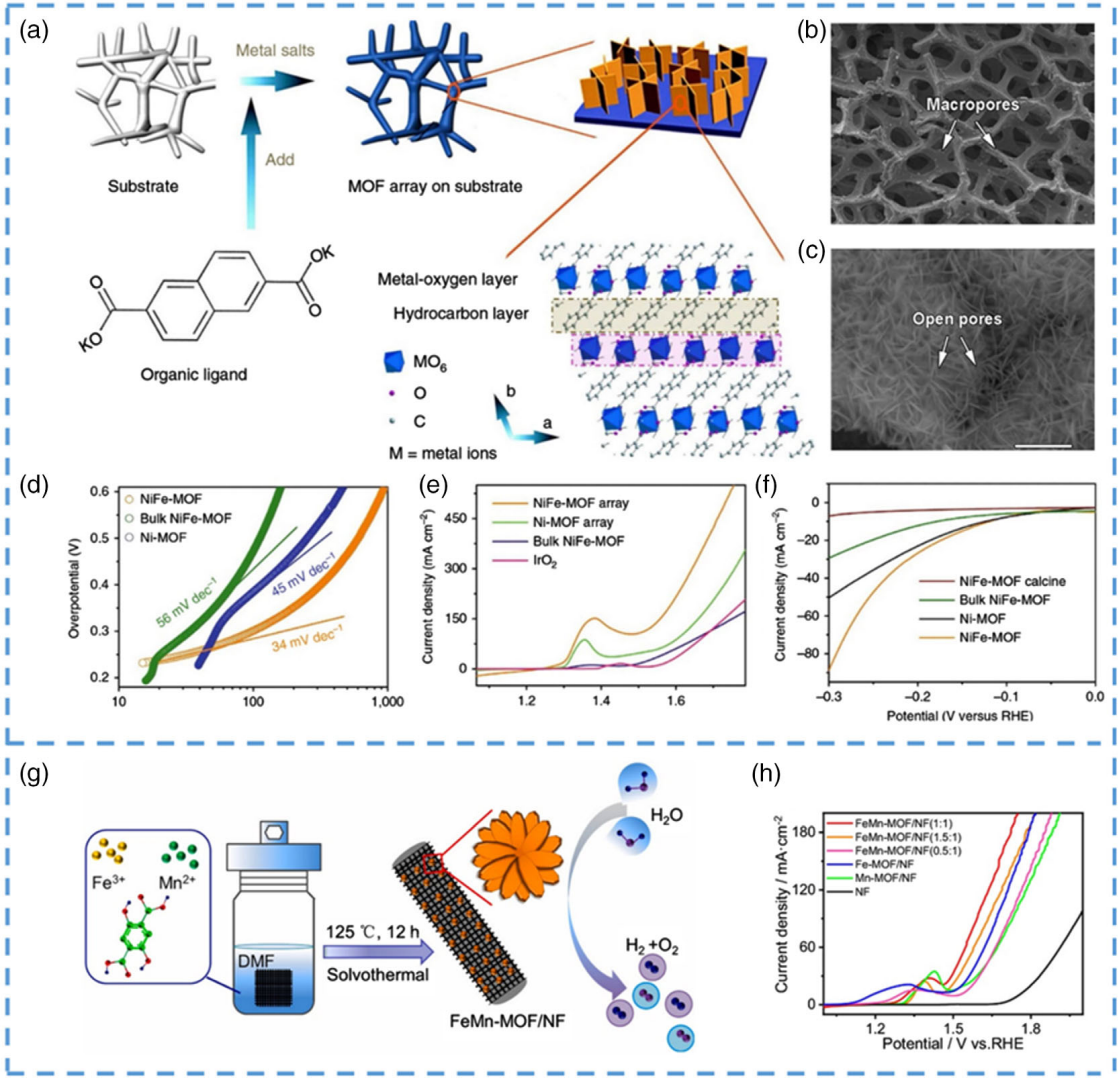
a) Synthetic process of MOF nanosheet array. b,c) SEM and TEM images of NiFe‐MOF electrode. d) Electrocatalytic properties of NiFe‐MOF and other samples for OER, Tafel plots obtained with NiFe‐MOF, Ni MOF, and bulk NiFe‐MOF. e) LSV plots obtained with NiFe‐MOF, nickel‐based MOF (Ni‐MOF), bulk NiFe‐MOF, and IrO_2_ for OER at 10 mV s^−1^ in 0.1 m KOH. f) Electrocatalytic properties of NiFe‐MOF and other samples for HER and overall water splitting, LSV plots obtained with NiFe‐MOF, bulk NiFe‐MOF, Ni‐MOF, and calcined NiFe‐MOF for HER at 10 mV s^−1^ in 0.1 m KOH. g) Schematic illustration of the fabrication procedure of FeMn‐MOF/NF. h) Fe‐MOF/NF, Mn‐MOF/NF, FeMn‐MOF/NF(1.5:1), FeMn‐MOF/NF(1:1), FeMn‐MOF/NF(0.5:1) electrochemical OER activities. a–f) Reproduced under the terms of the CC‐BY Creative Commons Attribution 4.0 International license (https://creativecommons.org/licenses/by/4.0).^[^ ^116^ ^]^ Copyright 2017, The Authors, published by Springer Nature. g,h) Reproduced with permission.^[^ ^118^ ^]^ Copyright 2021, Elsevier.

Duan et al. prepared ultrathin 2D nanosheets by simple growth of metal ions and organic ligands on the substrate by the chemical deposition method (**Figure** **11**a).^[^ ^116^ ^]^ The nanosheets are composed of two compounds, organic and inorganic hydrocarbons, which are connected at regular intervals. Through observation, it was found that these nanosheets always maintained transverse growth and their thickness remained unchanged at the initial thickness. Characterization confirmed that there were abundant pore structures inside the nanosheets (Figure 11b,c). These materials exhibit interesting properties in electrocatalysis, including a combination of highly exposed active molecular metal sites due to the ultrasmall thickness of the nanoflakes, enhanced electrical conductivity, and layered porosity. The catalytic performance of this nanoarray OER was tested with a small overpotential of 240 mV at 10 mA cm^−2^ and 20 000 s of stable operation with no detectable activity decay. According to the LSV, to better intuitively compare the advantages and disadvantages of catalytic activity, they also selected some existing OER catalysts as a reference (Figure 11d,e). It is clear that compared with other catalysts, NiFe‐MOF nanosheets exhibit the smallest overvoltage at the same current density. In steady‐state tests, the average numbers of electron revolutions and Faraday efficiency obtained in the rotating ring disk electrode show that the catalytic performance is good and oxygen can be produced successfully. At the same time, as a catalyst, its stability is also crucial, and the nanosheets produced have excellent stability, which has been demonstrated by various tests (such as cyclic voltammetry and electrochemical impedance). This nanosheet not only has excellent catalytic performance and stability of OER but also has a good application prospect for HER. In the water‐splitting reaction of the cathode, by polarizing the electrode to negative potential, its overpotential is also small compared with other catalysts, and its performance is not different from that of HER catalysis with precious metal (Figure 11f). The reasons for the optimization of its catalytic performance were analyzed. Although these are the catalytic active centers of OER and HER, their catalytic performance has not been fully demonstrated due to the limited number of exposed active sites. Nanoscale MOF materials can expose more active sites and exhibit better catalytic activity. In addition, many vacancies are formed on the 2D nanosheets, which provide a way for electron transport and greatly improve the conductivity of the material itself. Good conductivity means faster and smoother charge transfer within the nanosheets, which is one of the reasons for the better catalytic performance of the prepared nanosheets.

The hydrogen energy in clean energy comes largely from the preparation of hydrogen, which depends largely on water electrolysis. To get efficient and pure hydrogen from water electrolysis, some catalysts are added to the reaction. According to the steps of water electrolysis, it can be divided into HER and OER. Therefore, in order to improve the efficiency of water electrolysis, a large number of researchers devote themselves to the study of HER and OER catalysts.^[^ ^117^, ^119^, ^120^, ^121^, ^122^, ^123^ ^]^ The unique advantages of MOF material make it have great application potentials in the preparation of related catalysts. Guan et al. synthesized FeMn‐MOF crystals on NF by the one‐step method (Figure 11g).^[^ ^118^ ^]^ The nanoflower structure is more stable and less prone to structural collapse than other structures. They made several different materials by adjusting the molar ratio between the organic ligand and metal ions. When the molar ratio of metal ions to organic ligand mixture is 1:1, the crystals show a better nanoflower shape. In addition, the nanoflower shape, which increases the surface area of the crystal, exposes more active sites and improves its ability to catalyze the precipitation of oxygen and hydrogen (Figure 11h). Because of the direct correlation between the active area and the electrochemical double‐layer capacitance, they also tested the double‐layer capacitance, and the results showed that the optimal molar ratio of 1:1 was still the material with the maximum active area. The same method was used to test the MOF with both OER and HER activities, and the catalytic activity did not decrease significantly after 1000 cycles in the stability test. A three‐electrode system was constructed to decompose water by making them cathode and anode, respectively. At a voltage of 1.70 V, the current density maintained at 50 mA cm^−2^ is obviously lower than that of other systems, indicating that it has application value in water electrolysis applications. Among other things, they tested its performance in long‐term applications, leaving it for 60 days and again testing its ability to break down water. Under the same current density, the performance is not much worse than before, showing its repeatability and good cycle performance in practical applications. In general, this unique 3D nanoflower structure is more stable and less prone to collapse than previous 2D nanosheets, which is one of the reasons for its high catalytic activity over such a long period. The reasons for its high catalytic activity are as follows. First, the special nanostructure makes it easier to adsorb and generate oxygen, which improves the catalytic activity. On the other hand, due to nanocrystalization, the size is reduced and the morphology is changed, so the active site originally inside is exposed on the surface, which plays a synergistic effect with the two metal sites, which greatly improves its catalytic activity.

In addition to the increase of surface area and exposure of active sites, the nanocrystalline can fully disperse the precious metal on the crystal surface and improve its catalytic activity.^[^ ^124^ ^]^ Guan et al. used the impregnation method to refer to the precursor UiO‐66 and loaded Pb onto its surface.^[^ ^125^ ^]^ It showed superior activity in testing the activity of phenol hydrogenation. At 120 °C, phenol can be completely converted after 2 h, in addition to cyclohexanone also having high selectivity. Nano‐MOF materials have many fascinating advantages. Compared with the relatively single‐structure morphology of other materials, the structure of MOF materials itself has a variety of structures, including some common 12‐sided polygons and spheres. The MOF material is composed of metal ions and organic ligands, so it has the characteristics of both organic and inorganic materials. Most of the metal sites in the internal structure are asymmetric, and most of the pore sizes in the internal structure can be controlled, which contains a wealth of nanosized pore sizes, which brings more possibilities to the design of the internal structure of materials. The common design concept of using nano/microscale MOF materials as catalysts is to introduce more exposed metal sites in the interior, that is, to construct more coordination unsaturated bonds, in addition to creating some defect sites or connecting some organic ligands with catalytic activity. The nano‐MOF can effectively improve the diffusion limit caused by the size limitation, so the nano/microscale MOFs can maintain the catalytic performance while maintaining the stability of the structure. The use of MOF metal nodes and/or active sites on organic connectors makes sense because it enables on‐site separation of catalytic controlled components. In addition, while the good porosity of MOF is favorable for catalysis, the accessibility and mass transfer of the active site remain challenging, especially when catalytic reactions involve large substrates that tend to diffuse to the internal catalytic site. One of the ways to improve the diffusion of reactants and products in MOF materials is to prepare nano/microscale MOFs to lower the diffusion barrier. In the catalytic application of nano/microscale MOFs materials, the problem to be solved is their monodispersity. To solve this problem, many of the nano/microscale MOFs are directly synthesized into monodispersed particle sizes, but some of the nano/microscale MOFs are still crystallized in a polydispersed manner. Monodispersity and multidispersity have obvious influence on the catalytic effect. In the application of HERs introduced above, nano/microscale MOFs show good hydrogen evolution ability. In an alkaline solution, some MOFs containing azole and cyanide have strong interaction with the metal in the structure, which enable them to exist stably in the alkaline environment and also restricts the hydrolysis and condensation of the metal center. In the field of OER, nano/microscale MOF catalysts also have excellent performance, such as the wonderful nanoflower structure, which makes the active site in MOF expose to the maximum extent and greatly improves its original catalytic new energy. Nanocatalysts not only have many applications in the field of electrocatalysis but also show application potential in some common dehydrogenation and hydrogenation reactions. In addition to being a catalyst itself, there are also many nano/microscale MOFs which have been prepared as catalyst carriers, successfully dispersing some nanometal particles with catalytic activity fully in the catalytic environment, preventing their agglomeration to a certain extent. In summary, nano/microscale‐MOFs still have many potential applications in the field of catalysis.

### 3.2 Improved Energy Storage

Traditional energy consumption generally comes from fossil energy, but with the continuous progress of society, the reserves of fossil energy are becoming less, and the need for new energy storage technology is imminent.^[^ ^126^, ^127^, ^128^, ^129^, ^130^, ^131^, ^132^, ^133^, ^134^, ^135^ ^]^ Therefore, it is very urgent to develop efficient and reliable energy storage equipment, such as lithium–sulfur battery, lithium–oxygen battery, zinc–air battery, and supercapacitor. Compared with traditional technologies, these energy storage technologies are more convenient and efficient. In recent years, such energy storage technology has been widely used in computers, electric cars, and other applications. However, there are some challenges, such as maintaining stability after many cycles and reducing the original energy density and power ratio as the cell size increases. The combination of these electrode materials and nanotechnology can effectively address these challenges. The porous material and the high specific surface area after organization lead to the material exposing more active sites, which is very effective for the transfer of electrons in the material. The size of the active material particles plays a crucial role in the performance of lithium‐ion batteries (LIBs). Compared with microparticles, nanoparticles have more significant advantages in terms of fast charging capability, optimized power density, increased solid state and mass capacity, inhibition of memory effects, and increased cycle life. However, nanoparticles also have the disadvantages of low coulomb efficiency in the first period, complex manufacturing process, and high cost, which limit their commercialization. The unique internal structures of MOFs have great attraction in energy storage, but it still needs to face several difficulties.^[^ ^131^, ^132^, ^133^ ^]^ First of all, due to the poor electronic conductivity of MOF, the cycling property is very poor at high energy density.^[^ ^7^, ^8^, ^136^, ^137^ ^]^ Some MOF electrode materials have their own structures seriously damaged under multiple cycles. For example, when MOF‐177 is used in a lithium battery, the frame itself is irreversibly damaged after repeated cycles, and the material is crushed in the process.^[^ ^138^, ^139^ ^]^

MOF has been widely used in energy storage and conversion and can be generally divided into three types.^[^ ^31^, ^140^ ^]^ First, MOF is directly used to prepare electrode materials, which are directly used in energy storage and conversion. Second, it is combined with some other porous materials, such as carbon materials, polymers, and metal oxides, through the synergistic effect of the two to improve its energy storage and conversion capacity. The last one, taking MOF itself as a template, generates a new MOF derivative utilizing its unique structure. The MOF involved is also known as a “self‐sacrificing template,” resulting in the formation of metal oxides or porous carbon at a highly active site. Compared with the latter two methods, the preparation process is more complicated and costly, and some of the preparation conditions are more stringent. Therefore, MOF material directly used to prepare electrode materials has a broad application prospect. However, in order to expand its application potential, it is necessary to improve its electrochemical performance and cycle stability.^[^ ^137^, ^141^ ^]^ Therefore, improving the electronic conductivity of materials has become a key point to expand the potential of MOF materials in energy storage applications. More researchers are realizing that the size and shape of crystals inside energy storage materials affect energy storage and conversion. However, its shape and size can be effectively controlled by nanocrystallization. For example, changing the original large crystal to a smaller crystal size or changing the morphology of the original crystal will affect the movement and transfer of electrons inside the crystal. At the same time, it will weaken the original barrier of electron transmission and shorten the distance of electron transfer, which are conducive to improving its conductivity. The idea of using MOF to design energy conversion and energy storage equipment is mainly to prepare nano/microscale MOFs of different dimensions with high surface area and high porosity, so as to obtain electrode materials with more stable structure, more fully exposed active sites faster, and more convenient electronic transmission, so as to better realize energy storage and conversion.^[^ ^140^, ^142^, ^143^, ^144^, ^145^ ^]^

**Figure 12:**
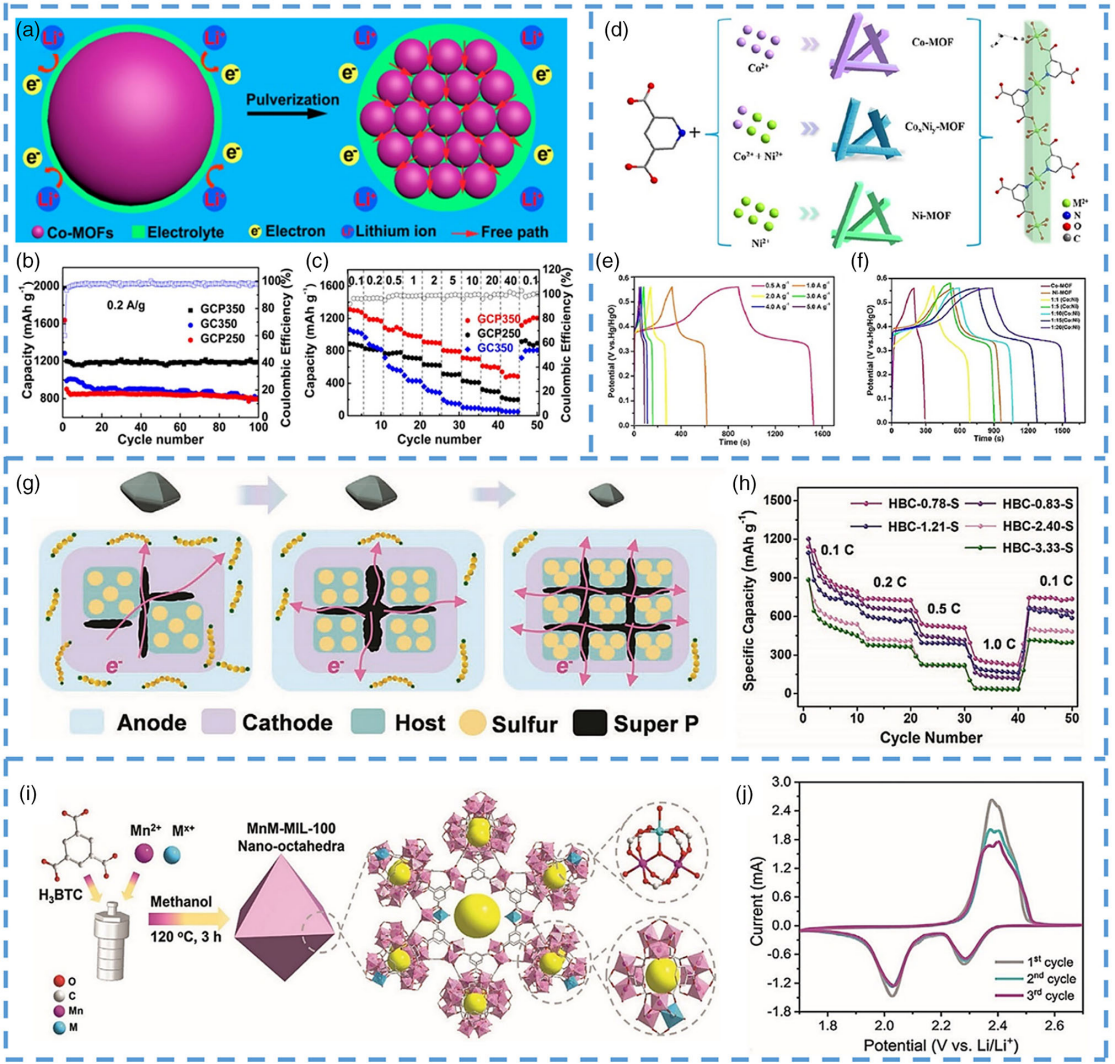
a) Schematic illustration of enhanced ion/electron transport in pulverized ultrasmall MOF nanocrystals. b) Cycling performance of GCP350, GCP250,and GC350 anodes at 0.2 A g^−1^. c) Rate performance of GCP350, GCP250, and GC350 anodes. d) Schematic illustration of the syntheses of Co‐MOF, Co_*x*_Ni_*y*_‐MOF (*x*/*y* = 1:1, 1:5, 1:10, 1:15, and 1:20), and Ni‐MOF. e) Galvanostatic charge/discharge (GCD) measurements of the Co‐MOF, Co_*x*_Ni_*y*_‐MOF, and Ni‐MOF electrodes at 0.5 A g^−1^. f) GCD profiles of the Co_1_Ni_20_‐MOF electrode at different scan rates and current densities. g) Diagram of sulphur content and lithium polysulfide of MIL‐96‐Al with different sizes as electrode materials during constant‐current charging/discharging process. h) Rate test for samples of different sizes. i) Schematic illustration of the general synthetic process for MnM‐MIL‐100 nano‐octahedra. j) CV curves of MnNi‐MIL 100 at a scan rate of 0.1 mV s^−1^. a–c) Reproduced with permission.^[^ ^150^ ^]^ Copyright 2018, American Chemical Society. d–f) Reproduced with permission.^[^ ^152^ ^]^ Copyright 2021, American Chemical Society. g–h) Reproduced with permission.^[^ ^153^ ^]^ Copyright 2022, Wiley‐VCH. i–j) Reproduced with permission.^[^ ^43^ ^]^ Copyright 2021, Wiley‐VCH.

In the early stage, Tang et al. controlled the size of synthesized UiO‐66 by lowering the temperature to prepare electrode materials for LIBs.^[^ ^146^ ^]^ Many researchers have found that with the reduction of the size, the resulting electrode material exhibits a higher specific capacity, proving that the nano‐MOFs could further improve the lithium storage performance of the obtained MOFs. The reduced nano UiO‐66 electrode material shortens the diffusion path of lithium ions, and greatly increases the diffusion frequency of lithium ion in the electrode material, thus improving the electrochemical performance.^[^ ^147^, ^148^, ^149^ ^]^ In addition, small particles will inevitably increase the specific surface area, which is the same as the optimization principle of catalytic performance introduced earlier. The increase in the reaction area will inevitably improve the performance of the material. However, in the above study, the cycling performance of the electrode material is yet to be strengthened after the test. In the third cycle, the peak area of the electrode material is increased compared with the area of the previous two cycles, indicating the formation of impurities. After several cycles, the shift of peak position also appeared in the CV diagram, leading to the appearance of overpotential. This is due to the poor electrical conductivity of UiO‐66. Subsequently, following the idea of reducing the size to improve electrochemical performance, researchers have carried out many studies and attempts. Xiao et al.^[^ ^150^ ^]^ prepared ultrasmall nano*‐*Co‐MOF and applied it in the anode of LIB, the ultrasmall nano*‐*Co‐MOF showing high electrochemical performance (**Figure** **12**a). First, Co‐MOF (about 100 nm in size) was successfully grown on graphene sheets using static electricity and coordination, and the large particles were pulverized into 5 nm ultrasmall crystals by heat treatment in a limited space. They evaluated the electrochemical performance of the ultrasmall crystals by conducting a constant‐current discharge–charge test of 0.01‐3 V at 0.2 A g^−1^. When the ultrasmall crystal prepared by nano*‐*Co‐MOF was used as the anode material of LIB, the initial discharge capacity was 1978 mAh g^−1^, and the reversible charging capacity was 1198 mAh g^−1^. The initial Coulomb efficiency was low, at 60.6%. However, the material showed good cycling performance, and the charge–discharge curve of the tenth time almost overlapped with that of the second time. In order to better test its cycling performance, a cycling performance test was carried out on it (Figure 12b). It is obvious from the figure that the initial capacity was 1201 mAh g^−1^, and the Coulombic efficiency (CE) was still more than 95% in the second cycle. After 1000 cycles, the capacity was still maintained at 1192 mAh g^−1^. For other materials without the addition of crushed nano*‐*Co‐MOF, their cycling performance is far superior to theirs. As a battery material, the battery not only needs stable cycling performance, but also needs high electronic efficiency to maximize the energy storage function of the battery. The team also tested its reaction rate as an anode material, using current densities ranging from 0.1 to 40 A g^−1^. They selected and tested the reversible capacity of the anode material made of the ultrasmall nanocrystals at different current densities of 0.1, 0.2, 0.5, 1, 2, 5, 10, and 20 A g^−1^. As shown in Figure 12c, the reversible capacity of this electrode is significantly higher than that of other anodes without nano/microscale MOFs, showing superior energy storage performance. It not only performs well at low current density but also exhibits a capacity of up to 494 mAh g^−1^ at a current density of up to 40 A g^−1^, indicating that the structure of the electrode material is not easy to change to affect the performance. In addition, the team tested other materials that did not contain ultrasmall Co‐MOF nanocrystals, again demonstrating that the superior electrochemical performance is due to the involvement of nano‐MOF. The crushing of large particles, namely nanocrystallization, greatly increases the contact area between the active substance and the electrolyte. In addition, the reduction of nanoparticle size greatly increases the charge transfer rate and increases the diffusion of lithium ions in the nanoparticles. The combination of the two greatly improves the reaction conditions. The energy storage performance of anode of LIBs is superior to that of other MOF‐based electrode materials, which is brought about by nanocrystallization and proves the necessity and practicability of MOF nanocrystallization.

In addition, the miniaturized nanostructure of MOF not only affects the application of lithium batteries, but also has a profound impact on the use of MOF as electrode material for supercapacitors.^[^ ^151^ ^]^ The increasing demand for energy storage requires more electrochemical energy storage devices. The reasonable design and construction of novel electrode materials to improve the performance of energy storage devices have become an important topic under the current new energy development strategy and also an important guarantee to promote the sustainable development of social economy. As a promising electrochemical energy storage device, hybrid supercapacitors organically combine the energy storage advantages of batteries and conventional supercapacitors and realize the integration of high energy storage and high power output. As an important part of the hybrid supercapacitor, battery electrode has a decisive influence on the energy storage performance of the whole device. By regulating the content of Ni^2+^ in MOF, Hang et al. obtained Co‐MOF and Co_*x*_Ni_*y*_‐MOF with different diameters (X and Y represent the mole ratio of cobalt and nickel, respectively) of Ni‐MOF nanorods (Figure 12d).^[^ ^152^ ^]^ Ni‐MOFs have excellent electrochemical performance due to their conjugated bonds, which result in the electrochemical reaction process of Ni‐MOF with good permeability, low resistance, and rapid ion transfer in the electrolyte. By characterizing the three MOF with different Ni^2+^ content, they found that all were composed of homogeneous nanorods, but the size of nanorods gradually decreased from 800 nm to about 300–400 nm with the increase in Ni^2+^ ions, indicating that the team successfully regulated and controlled the size of MOF. Electrochemical tests were carried out on nanorods obtained at different mole ratios. When Co:Ni = 1:20, the electrode material at this time has the most specific capacitance (as shown in Figure 12e). The constant‐current charge–discharge curve in Figure 12f is almost symmetrical, which proves that Co_1_Ni_20_‐MOF has good electrochemical performance when used as the electrode material for supercapacitors. The charge–discharge curves obtained at different current densities show that the capacitance retention of the electrode material decreases with the increase of current density. However, Co_1_Ni_20_‐MOF still maintains the highest discharge efficiency. The team found that the electrode material for supercapacitors obtained at the optimal mole ratio had high specific capacitance, good rate performance, and long‐term cycle stability. The synergistic effect of the nanorods with smaller diameter and cobalt and nickel elements improves the original reaction kinetics, resulting in excellent supercapacitor performance of the prepared electrode materials. The nanosized particles not only affect the electron movement but also affect the inhibition of sulfur shuttle effect in lithium–sulfur battery, thus improving the energy storage performance of lithium–sulfur battery.^[^ ^154^, ^155^, ^156^, ^157^, ^158^ ^]^ Early in 2014, Zhou et al. prepared different sizes of Zn‐ZIF‐8 ranging from 15 nm to 2 μm and applied them to lithium–sulfur batteries respectively and tested their electrochemical performance.^[^ ^49^ ^]^ The team found that as the nanocrystals shrank in size, their specific cyclic capacity increased. Cai et al. also prepared nano‐Cu‐TDPAT of different sizes for lithium–sulfur batteries, and the test results showed that the smallest particle size had the largest capacity.^[^ ^159^ ^]^ In lithium–sulfur batteries, the most important thing to pay attention to is sulfur fixation to prevent irreversible loss of sulfur during charge and discharge. Early results suggest that MOF materials are suitable for lithium–sulfur batteries, and a number of studies have been carried out in recent years. More recently, Geng's research has shown that MOFs with different particle sizes have different adsorption capacities for lithium polysulfide, which directly affect its performance in lithium–sulfur batteries.^[^ ^153^ ^]^ MIL‐96‐Al, one of the 3D MOF materials, was used in this study to obtain crystals with different shapes and sizes (e.g., hexagonal flake, hexagonal biconical, and hexagonal prismatic biconical) by adjusting the proportion of solvents involved in the reaction. The results show that the smaller the grain size, the faster the electron transfer speed, the higher the stability, and the higher the sulfur load, as shown in Figure 12g. From Figure 12h, it is obvious that the smaller the sample size, the better the rate performance. Not only that, the resistance of a smaller sample will also be reduced. It can be seen that size has such a significant impact on the performance of the energy storage materials in lithium sulfur batteries. Li et al. prepared MIL‐100 nanooctahedron containing multiple metals, which was used as the cathode of the lithium–sulfur battery (Figure 12i).^[^ ^43^ ^]^ Monometal, bimetallic, and trimetallic nano‐MIL‐100 were prepared by the simple hydrothermal method. This unique nano‐octahedral structure, with adjustable unsaturated metal bonds and neatly arranged voids, is very helpful to prevent excess sulfide in lithium–sulfur batteries. In the cyclic voltammetry test curve (Figure 12j), the first two distinct cathode peaks in the CV curve of MnNi‐MIL indicate conversion from S_8_ to long‐chain polysulfide. In subsequent tests, it showed better cyclic stability and higher capacity retention rate. This unique nano‐octahedral structure made electrons encounter less resistance when transferring. In this study, it was also clearly proposed that the nanostructure would make the material have excellent electrochemical performance. In addition, shape has a significant effect on the adsorption of lithium polysulfide. Based on this, Li's team used polyhedral nanostructures to improve its sulfur‐fixation ability.^[^ ^160^ ^]^

Inspired by the above studies, rather than just preparing individual nanocrystals, researchers began to prepare nanocrystal arrays using small particles to obtain nano/microscale MOFs structures that are more conducive to electron mobility. Li et al. pioneered the synthesis route of MOF nanowire arrays to prepare solid supercapacitor electrode and finally found that the performance of the prepared electrode material was not much different from that of carbon materials.^[^ ^161^ ^]^ Yaghi et al. grew Cu‐CAT on carbon fiber paper and could control its length and diameter by adjusting the reaction time.^[^ ^10^ ^]^ Cu‐CAT is a MOF material with good electrical conductivity. Through the structure characterization of nanowire array, it was found that the prepared nanowire was a hexagonal prism shape, with a regular hexagonal top surface, and covered the whole fiber surface along the direction of [001]. This structure also facilitates the transmission of electrons and provides conditions for rapid charge and discharge. This was also proved in the subsequent electrochemical performance test. The CV diagram and constant current charge and discharge showed that the electrode material prepared by the nanowire array had good stability, and its specific capacitance could still maintain 66% under the condition of large current density. After 5000 cycles, 88% of the initial capacitance can still be maintained, which shows high stability compared with other MOF‐based electrode materials. In order to more clearly compare the advantages brought by the meter wire array, the team synthesized Cu‐CAT powder electrode in the same way, maintaining the same test conditions to compare its electrochemical performance. It was found that the capacitance of the electrode prepared by nanowire array was much larger than that of the powder Cu‐CAT electrode, nearly twice as much as that of the powder electrode, and its impedance was much lower than that of the powder electrode. The reason for this is that the powdered MOF is attached to the surface by a binder, which increases the resistance of ion transport. On the contrary, the nanowire array MOF grows directly on the surface, which reduces the resistance to some extent. This nanostructure greatly reduces the resistance on the surface of the original electrode and electrolyte, as well as the resistance of charge transfer within the electrolyte, which greatly increases the ion exchange and transmission between the active substance and electrolyte, thus achieving high electrochemical activity and opening up the application of special nanoarray structures in the field of energy storage.

**Figure 13:**
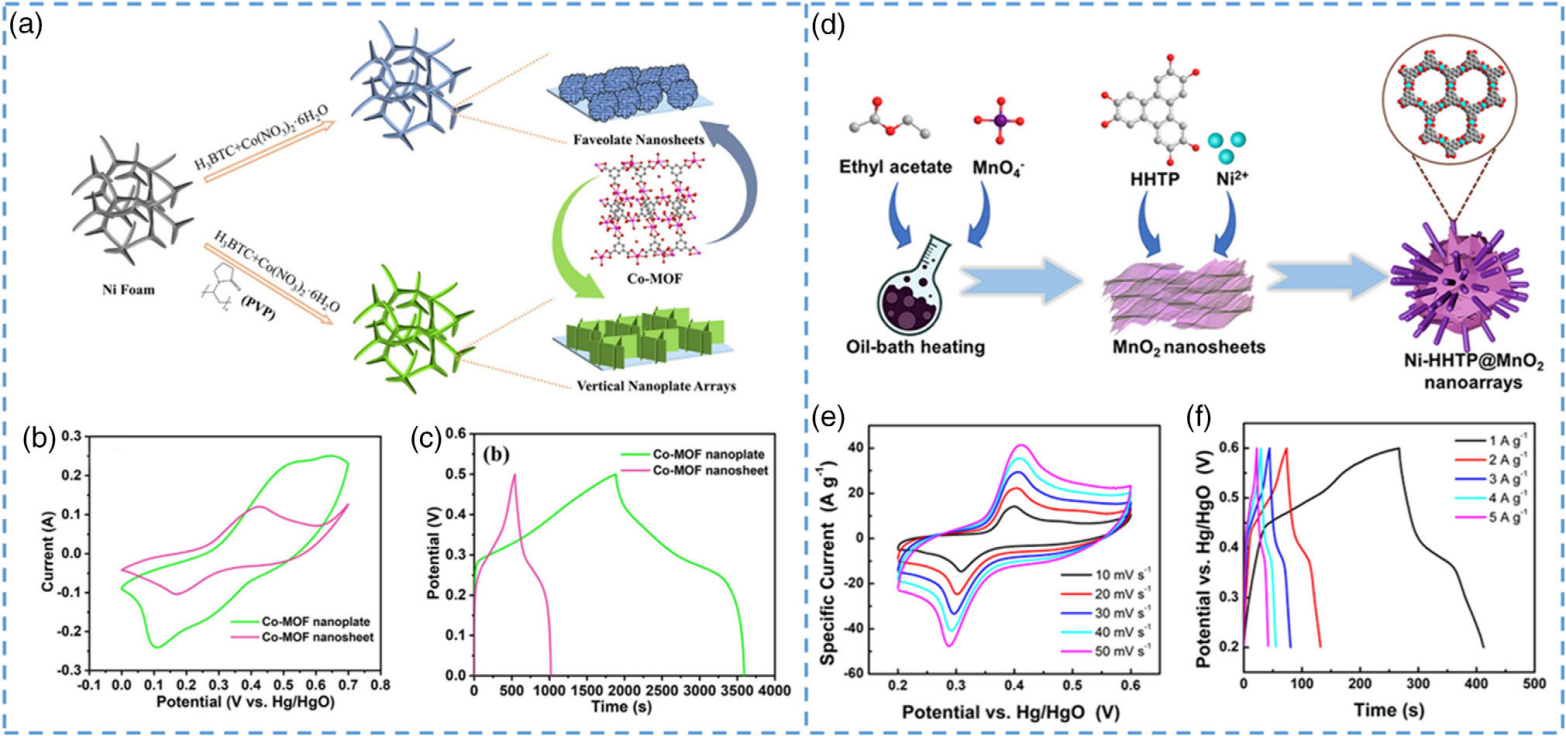
a) Schematic illustration of the synthesis of faveolate Co‐MOF NS and Co‐MOF NP arrays. b) CV curves and c) GCD curves of the Co‐MOF NP arrays and faveolate Co‐MOF NS electrodes at 10 mV s^−1^ and 5 mA cm^−2^. d) Schematic illustration of the formation of MnO_2_@Ni‐HHTP nanoarrays. e) CV curves at scan rates of 10–50 mV s^−1^ of NHMO‐5 at a voltage window of 0.2‐0.6 V in 3.0 m KOH. f) GCD curves of NHMO‐5 with current densities of 1.0–5.0 A g^−1^. a–c) Reproduced with permission.^[^ ^67^ ^]^ Copyright 2021, Elsevier. d–f) Reproduced with permission.^[^ ^162^ ^]^ Copyright 2021, American Chemical Society.

Some early studies on the preparation of electrode materials using nanoarray structures have indeed improved their energy storage performance to a certain extent, but in general, their performance and stability still need to be improved. With the in‐depth research on this structure, the performance of some electrode materials has been far higher than their previous performance. By adjusting the concentration of polyvinylpyrrolidone, Zhang et al. successfully prepared a 2D nanoplate array Co‐MOF on the surface of nickel foam and applied it in the field of energy storage.^[^ ^67^ ^]^ The team synthesized the Co‐MOF by one‐step solvothermal method, adjusted the shape of the synthesized MOF by adding PVP, and finally synthesized nanoplates and 2D nanoplate arrays perpendicular to the nickel foam. The morphology of MOF without PVP was characterized by scanning electron microscopy, and it was found that they were grown in situ and superimposed together with nickel foam as support, and finally presented on the surface as microspheres. The microspheres observed on the surface were mainly composed of Co‐MOF nanosheets (**Figure** **13**a) and were labeled Co‐MOF NS. In order to obtain a better nanostructure, surface agents with hydrophobicity were added to effectively control the original agglomeration and influence growth dynamics, and finally the nanosheet array structure with higher surface area was formed, which was labeled Co‐MOF NP. Through characterization, it is found that elements C, O, and CO are uniformly distributed in the crystal. Figure 13b,c shows the CV diagrams of two different morphologies of nano/microscale‐MOFs tested at 10 mV s^−1^ current density. It can be seen from the figure that Co‐MOF NS only has a pair of redox peaks, while Co‐MOF NP has two redox peaks, indicating that the activity of the nanosheet array is superior to that of the concentrated nanosheet. Other tests have also proved that nanosheet arrays have better application potential in energy storage devices. More Co active sites were exposed in the nanosheet array than in the microsphere, which resulted in a significant optimization of its electrochemical kinetics. In the impedance test, the nanosheet array also has a low internal resistance because the nanosheet perpendicular to the surface has more controllable charge transfer paths, so the diffusion of charge in it is more effort‐saving, greatly reducing the moving barrier, whereas the agglomeration of microspheres is exactly the opposite, which also confirms the necessity of nanocrystallization. In the cycle performance test, Co‐MOF NP can maintain 84% capacity after 5000 cycles, 10% higher than Co‐MOF NS. After several cycles, the original concentrated microspheres were destroyed, while the vertical nanosheets were not affected much. So the team believes that the nanosheet array of MOFs has good potential in the direction of energy storage. In addition, not only nanosheets but also polyhedral nanostructures can also have excellent electrochemical properties. The increasing demand of energy storage requires more electrochemical energy storage devices. As a promising electrochemical energy storage device, hybrid supercapacitors organically combine the energy storage advantages of batteries and supercapacitors and realize the integration of high energy storage and high power output. As an important part of hybrid supercapacitor, battery electrode has a decisive influence on the energy storage performance of the whole device. Therefore, not only the team believes that the MOF of nanocrystal arrays has good potential in energy storage, but other teams also agree with this view and study the application of nanocrystal arrays in supercapacitors. Duan et al. fabricated conductive MOFs with nanoarrays of different lengths and explored the electrochemical performance of the prepared positive material in supercapacitors by combining it with transition metal oxides (Figure 13d).^[^ ^162^ ^]^ Compared with other pristine MOFs, the graphene structure of conductive MOF is similar to that of in‐plane π bond and generally presents a 2D structure. The unique 1D cylindrical channel makes the ion transfer more convenient, thus improving its electrical conductivity. It can be seen from the cyclic voltammetry curve in Figure 13e that NHMO‐5 has an obvious redox peak with a large redox peak area, indicating that NHMO‐5 has the potential to be applied in supercapacitors, which is also confirmed by the constant‐current charge–discharge test in Figure 13f. The team used a series of tests to demonstrate that the unique nanoarray structure exhibits excellent energy density and rate performance as well as good cycling performance in a water‐based asymmetric supercapacitor with an outstanding specific capacity of 368.2 F g^−1^ at 1 A g^−1^. In fact, the relatively thin MnO_2_ provides a wealth of redox active sites, and not only that, the Ni‐HHTP nanorods arrays grown in situ on the surface of MnO_2_ provide ample space for ion transfer, thus ensuring improved electrical conductivity.

The application of pristine MOF in energy storage has been limited due to its poor electrical conductivity and the destruction of the porous structure in some electrolytes with increasing cycle time. Compared with the pristine MOF, nano/microscale‐MOFs have a larger surface area and a smaller size, giving them the possibility of being more easily combined with other materials. Using this advantage, it is very conducive to the preparation of materials for energy storage devices, such as solar cells, batteries, and supercapacitors. In recent years, nano MOF has been regarded as a promising electrode material. When they are used in electrode materials, the abundant pore structure in their interior can provide space for the volume increase caused by the ionic liquid‐type electrolyte when they are extruded and embedded. Moreover, the rich porous structure can greatly improve the contact rate between the electrode material and the electrolyte, which also reduces the diffusion path of ions in the electrolyte to the electrode material and can also bring more active sites. In recent years, some nanostructures with unique structures also show broad application prospects in energy storage and transformation. For example, some unique hollow nanospheres and some nonspherical structures have a lot of voids, which can effectively eliminate the volume change of electrode materials in the charging and discharging process, so as to extend the service life of energy storage equipment to a certain extent. A typical example is LIBs, which have more mature technology and higher energy storage performance than other types of metal‐ion batteries. With repeated charging and discharging, a large number of dendrites are formed on the surface of the lithium electrode, which greatly hinders the transfer of ions. When there is no room for the movement of ions, the batteries will not work properly. However, enough space should be left to extend the service life to a certain extent. In lithium–sulfur battery, the amount of sulfur load directly affects the performance of LSBs, which has become a problem to be solved on the way to obtain high‐performance LSBs. nano/microscale‐MOFs can effectively solve this problem. Studies by some researchers have proved that MOFs with smaller sizes have a larger sulfur load than that with larger sizes, which makes lithium–sulfur batteries prepared with nano/microscale‐MOFs have better energy storage performance. In addition, enriched active sites are also exposed due to nanoprocessing. Compared with the mesoscale MOF as electrode material, the nanocrystalline MOF has better stability and catalytic activity.^[^ ^47^, ^163^ ^]^ However, from medium size to small size, from large size to small size, the surface area of the crystal also increases, increasing the chance of reaction on the surface and increasing the reaction area, which are the reasons for the optimization of the performance.^[^ ^164^, ^165^ ^]^

### 3.3 Others

MOFs also have great potential in sensing applications as alternatives to metal oxides, nanoparticles, and quantum dot‐based materials.^[^ ^166^, ^167^, ^168^, ^169^, ^170^ ^]^ In the study of Rieter et al., the application potential of nano/microscale‐MOFs as a nuclear magnetic resonance contrast agent was confirmed.^[^ ^171^ ^]^ The team prepared Gd‐MOF nanorods with a length of 100–1000 nm and a diameter of 40–100 nm by the microemulsion method, which was used as an anode comparator to compare the relaxation of the nanorods with the Gd‐containing colloids, and found that the relaxation of the nanorods was fully 10 times higher than that of the colloids. Moreover, the relaxation value of the nanorods is also the highest due to the higher Gd‐bound water exchange rate, which caused by the high surface area of the nanoparticles. In addition, MOFs with unique luminescent properties and porous structures can be designed and modified to become recognition points, so they can be widely used in light‐related sensing fields. Compared with other sensing materials, the crystallinity of MOF is higher, and the internal structure of nano MOFs can be obtained by simple single‐crystal diffraction, and now many nano/microscale‐MOFs are directly used in fluorescence sensors. Ren et al. synthesized bulk MOF and nano/microscale MOFs by the hydrothermal method and ultrasonic‐assisted method, respectively, and folic acid was used as the target molecule to compare the difference in fluorescence properties of the two MOFs.^[^ ^172^ ^]^ As an insoluble solid particle, MOF applied in the fluorescence sensor is generally dispersed in the state of suspension in the fluorescence solvent. Their particle size will directly affect the dispersion and sedimentation rate of MOFs in solution and then affect the accuracy of the signal and precision of the detection method. In the team's test, the fluorescence intensity of large‐particle Tb‐MOF decreased rapidly in a very short time, while the nano‐Tb‐MOF still maintained a relatively stable state within 60 min, with good precipitation resistance. Nano‐Tb‐MOF is much more sensitive to fluorescence tests using folic acid as the target molecule than bulk ones. Xia et al. synthesized ZIF‐8 as the MOF scaffold to encapsulate two types of size‐matching fluorescent molecules.^[^ ^173^ ^]^ By adjusting the encapsulation mode and concentration, different fluorescent‐emitting materials ranging from blue to red including white were obtained. In addition to ZIF‐8, UiO‐66‐NH_2_ also possesses unique fluorescence properties. Wu et al. synthesized fluorescent MOFs@MOFs with stable UiO‐66‐NH_2_ as the core substrate, which displayed sensing selectivity for Hg^2+^ ion.^[^ ^174^ ^]^ In conclusion, the size of MOF is closely related to its fluorescence performance, and nano‐MOF is more suitable for fluorescence sensor. In addition to the traditional sensor application, it can also be used as a biological detection probe. Ma et al. used nano/microscale MOF as a novel heterogeneous fluorescent probe for in situ detection of hydrogen sulfide gas and completed confocal imaging in living cells.^[^ ^175^ ^]^ Due to the porous structure of MOFs itself, the original characteristics become more significant after being nanosized, which is very conducive to introducing responsive site ions (such as some metal ions) into the channel. The team chose to introduce Cu(II) metalated into the MOF as the metal active center, as the point that can respond to hydrogen sulfide gas. Due to its high porosity, large specific surface area, interaction between metal and organic ligands, and abundant active sites, the MOF has various advantages of nanomaterials after nanomination, such as effectively avoiding probe extravasation and increasing probe retention and enhancing probe endocytosis ability. It makes the probe made with nano/microscale MOFs biocompatible and can respond quickly in situ when detecting hydrogen sulfide gas. Compared with the probes prepared from other materials, this probe has higher principle and is nontoxic, which is very conducive to the detection of hydrogen sulfide at physiological PH.

**Figure 14:**
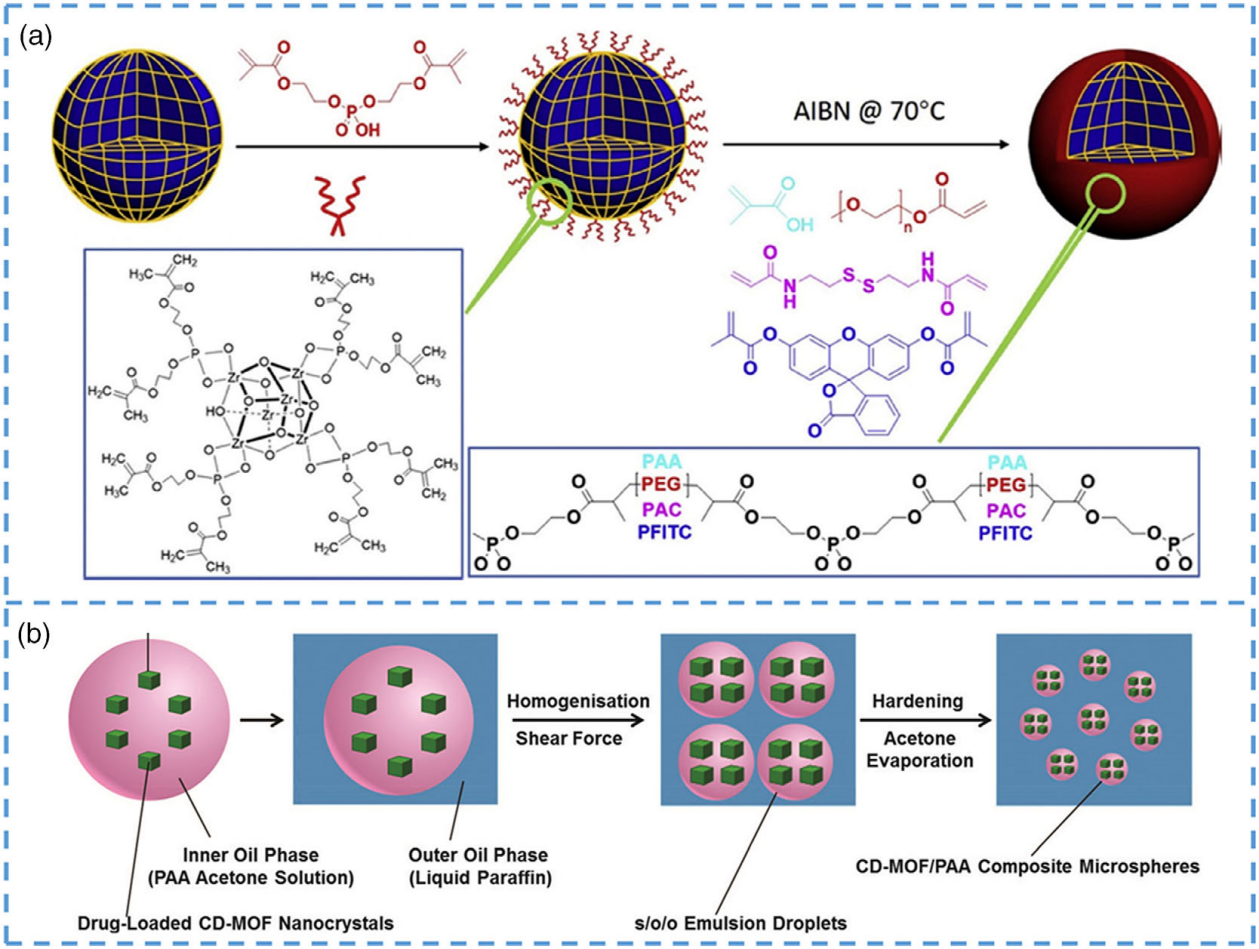
a) Schematic diagram of in situ polymerization on MOF nanoparticles. b) Schematic diagram of preparation process of Cd‐MOF/PAA composite microspheres. a) Reprodued with permission.^[^ ^182^ ^]^ Copyright 2019, Elsevier. b) Reproduced with permission.^[^ ^183^ ^]^ Copyright 2017, Royal Society of Chemistry.

In recent years, researchers have found that nano/microscale‐MOFs are very beneficial as a nontoxic drug‐carrying nanocarrier, which can overcome many of the challenges faced by traditional nanocarriers.^[^ ^176^, ^177^, ^178^, ^179^, ^180^ ^]^ Early researchers synthesized MIL‐MOF with a diameter of less than 200 nm by solvothermal method as a nanocarrier. In this study, it was shown that nano‐MIL‐MOF can absorb about one‐quarter of the anticancer drug busulfan, which is 6 times of the absorption of other types of polymer carriers and 60 times of lipids, showing good drug loading capacity.^[^ ^53^ ^]^ Second, the nano/microscale‐MOF carrier did not enter human organs in the subsequent rat toxicity test but was excreted with feces. In oral drugs, new ways of controlling release are also needed, and more efficient delivery systems are designed to induce drugs to reach the body precisely. Javanbakht et al. prepared anticancer drugs into solvents and encapsulated them in Zn‐MOF‐5 and designed a new safe drug delivery method.^[^ ^181^ ^]^ Liu et al. prepared nano/microscale MOFs with high physiological stability and hydrochloride stimulation response for intravenous therapy of tumors (**Figure** **14**a).^[^ ^182^ ^]^ It has been shown in studies that the nanoparticles can achieve stimulus‐responsive drug delivery with high stability and intracellular drug release. These results indicate that MOF nanoparticles can be used as an efficient and safe drug delivery platform in the future. Li et al. established an improved hydrothermal process to efficiently produce micrometers (5–10 μm) and nanometers (500–700 nm) of Cd‐MOF particles with uniform size, smooth surface, and powdery appearance (Figure 14b).^[^ ^183^ ^]^ Cd‐MOF nanoparticles prepared by the hydrothermal method can trap insoluble and unstable ibuprofen and lansoprazole in nanoparticles by cocrystallization. This method is more efficient than traditional impregnation, with a larger drug load and without the damage of the carrier structure during drug release. In the study of Zhou et al. study, nano‐Pd‐MOF can not only store a large amount of highly reduced hydrogen, but also be used in tumor‐targeted photoacoustic imaging.^[^ ^184^, ^185^ ^]^ Isabel et al. loaded three drugs into UiO‐66 by exploiting a defect in it.^[^ ^186^ ^]^ The one‐pot method closely combines the desired drugs with the coordination defect but still endows some pores, providing an opportunity for the fourth drug payload to enhance the activity of antibreast cancer cells.

Through these advances, nano/microscale‐MOFs will emerge as a promising new functional nanomaterial with the potential for significant catalytic, energy storage, and pharmaceutical applications, and **Table** **1** lists the relevant studies involved in the article. It is clearly demonstrated that nano/microscale‐MOFs not only improve catalytic capabilities, expand the efficiency of energy conversion and storage, but also have the potential for far‐reaching applications in areas such as sensors, fluorescence, and drug delivery, but still have a long way to go.

**Table 1:** Application of modified MOFs

Materials	Synthetic method	Applications	Performance	Ref.
UiO‐66	Hydrothermal reaction	LIBs	Reversible capacity: 118 mAh g^−1^	[146]
Co‐MOF	Pulverization method	LIBs	Reversible capacity: 1301 mAh g^−1^ Cyclic stability: 98.6% at 10 A g^−1^ after 2000 cycles	[150]
ZIF‐8	Mechanochemical synthetic	LSBs	Discharge capacities: 1055 mAh g^−1^ Cyclic stability: 100% after 300 cycles.	[49]
MIL‐96‐Al	Controlled mediation method	LSBs	Discharge capacities: 1083 mAh g^−1^ Cyclic stability: maintain 448 (200 cycles)	[153]
MnM‐MIL‐100	Hydrothermal reaction	LSBs	Discharge capacities: 1579.8 mAh g^−1^ Cyclic stability: maintain 708 (200 cycles)	[43]
Zn‐MOF‐74	Controlled mediation method	PIBs	Reversible capacity: 595.8 mAh g^−1^ Cyclic stability: 150.7 mAh g^−1^ at 2.5 A g^−1^ after 400 cycles	[64]
Co‐MOF	Controlled mediation method	SCs	Specific capacitances: 17.9 F g^−1^ at 10 mA g^−1^ Cyclic stability: 88.0% after 3000 cycles	[67]
Co‐MOF	One‐pot method	SCs	Specific capacitances: 1020 F g^−1^ at 0.5 A g^−1^ Cyclic stability: 96.7% after 5000 cycles	[41]
Ni‐MOF	Microwave radiation method	SCs	Specific capacitances: 979 F g^−1^ at 0.5 A g^−1^ Cyclic stability: 98% after 5000 cycles	[103]
NiCo‐MOF	Hydrothermal reaction	SCs	Specific capacitances: 530.4 F g^−1^ at 0.5 A g^−1^ Cyclic stability: 100% after 2000 cycles	[104]
Cu‐CAT	Interfacial growth approach	SCs	Specific capacitances: 120 F g^−1^ at 0.5 A g^−1^ Cyclic stability: 80% after 5000 cycles	[161]
CoNi‐MOF	Hydrothermal reaction	SCs	Specific capacitances: 59.7 F g^−1^ at 0.5 A g^−1^ Cyclic stability: 93.59% after 4000 cycles	[152]
C‐MOF	Controlled mediation method	SCs	Specific capacitances: 368.2 F g^−1^ at 1 A g^−1^ Columbic efficiency: 95.4%	[162]
CoFe‐MOF	Template method	OER	$\eta_{\text{10}\;{\text{mA/cm}}^{2}}$ = 176 mV in 1.0 m KOH Tafel slop: 35.1 mV dec^−1^	[73]
FeNi‐MOF	One‐pot method	OER	$\eta_{\text{100}\;{\text{mA/cm}}^{2}}$ = 266 mV in 1.0 m KOH Tafel slop: 52.4 mV dec^−1^	[79]
NiCo‐MOF	One‐pot method	OER	$\eta_{\text{10}\;{\text{mA/cm}}^{2}}$ = 256 mV in 1.0 m KOH Tafel slop: 81.8 mV dec^−1^	[81]
NiFe‐MOF	Chemical bath deposition method	OER,HER	OER:$\eta_{\text{10}\;{\text{mA/cm}}^{2}}$ = 240 mV in 1.0 m KOH Tafel slop: 34 mV dec^−1^ HER:$\eta_{\text{10}\;{\text{mA/cm}}^{2}}$ = 134 mV in 1.0 m KOH	[116]
FeMn‐MOF/NF	Hydrothermal reaction	OER,HER	OER:$\eta_{\text{50}\;{\text{mA/cm}}^{2}}$ = 290 mV in 1.0 m KOH Tafel slop: 87.02 mV dec^−1^ HER:$\eta_{\text{50}\;{\text{mA/cm}}^{2}}$ = 260 mV in 1.0 m KOH Tafel slop: 156.6C mV dec^−1^	[118]
FeCo‐MOF	Template method	ORR	Current density: −0.0601 A cm^−2^ *i*_0_: 29.59 × 10^−4^ A cm^−2^	[74]
Ni‐MOF@NiO/Ni	Solvothermal	UOR	$\eta_{\text{10}\;{\text{mA/cm}}^{2}}$ = 170 mV in 1.0 m KOH Tafel slop: 48.1 mV dec^−1^	[115]
NTU‐9	Top‐down method	Sensing	/	[88]
HSB‐W5	Top‐down method	Sensing	/	[91]
TMU‐23	Microwave radiation method	Sensing	/	[65]
CuBDC MOF	Interfacial growth method	Gas separation	/	[87]
Ni*‐*Co‐MOF	Controlled mediation method	Microwave absorption	/	[66]
Ni‐MOF	One‐pot method	Catalyzing N_2_H_4_	/	[80]
ZIF‐8/ZIF‐67	Template method	/	/	[72]
Cu‐BTC MOF	Interfacial growth method	/	/	[95]
HKUST‐1	Interfacial growth method	/	/	[97]
Th‐MOF	Microwave radiation method	/	/	[101]

## 4 Conclusion and Outlook

Herein, we summarized the recent advances in synthetic strategies to prepare nano/microscale MOFs with controllable structures. Based on these advances, nano/microscale‐MOFs will emerge as a promising new class of functional nanomaterials with the potential to significantly impact the fields of catalysis, energy storage, and pharmaceutical applications. Based on the above, this review focuses on the progress of the efficient preparation of nano/microscale‐MOFs through synthetic strategies such as controlled mediation, template, and one‐pot methods. This type of material increases the contact area between reactants, exposing the internal active site more fully, and it also allows electrons to be transferred internally through a smaller barrier, enabling more efficient transport of ions between the electrode material and the electrolyte. These are urgently needed in the field of energy storage and electrocatalysis. Although today's nano/microscale MOFs preparation technology and applications have matured, it is believed that future developments will be more exciting by improving the following issues. 1) This review introduces a variety of methods that can be used for the synthesis of nano/microscale MOFs, but none of them is perfect and each has its own limitations. Although controllable regulation method can be used to change some conditions (such as reaction time, reactant volume ratio, reaction time) to obtain crystals of different morphs and sizes, however, in order to control the size and shape of the synthesized MOF more accurately, the reaction conditions need to be tested several times and the system should be optimized. The template method avoids the tedious process of systematic optimization of reaction conditions and accurately controls the particle size using templates with different diameters. It is often combined with controlled adjustment methods to achieve precise size control, not only particle size but also length. Compared with the traditional heating method, the microwave radiation method is more rapid and convenient and greatly reduces the reaction time. Obviously, by optimizing the size of MOF and controlling the size of synthesis through means, the unique properties of small size can be maximized in various fields. However, there are still many challenges on how to control the size and morphology more accurately. At present, some methods to reduce the size are only used in small‐scale experiments, and the efficiency is not high and it takes a long time to consume. Further research should focus on developing more efficient and scalable methods for downsizing. 2) Although MOFs have many desirable advantages, such as porous structure, large specific surface area, and controllable chemical composition, it is known that the porous structure and surface area of the MOF slow down the volume consumption of the electrode material when it reacts with the electrolyte in battery, thus extending the service life of the electrode materials. However, this is not the case in practical applications. Higher porosity will reduce the energy density of electrode materials, which is only one aspect. Compared with some common carbon materials, in some special solution environment, such as common alkaline electrolyte, with the delay of time, its stable structure will be accompanied by collapse or even collapse. Other researchers have made a lot of efforts, but it is still an urgent problem to be solved in many application fields. 3) Research on the design of nano/microscale MOFs composites should be strengthened. Some conductive metals and metal oxides were introduced into MOF, including some precious metals with high active sites, to improve its conductivity. However, precious metals are relatively expensive, which is not conducive to large‐scale investment in practical applications. In addition, some researchers also chose to retain the excellent frame structure of MOF itself at high temperature and assemble it with carbon materials with high conductivity. Part of this method requires more stringent reaction conditions, such as higher temperature or pure calcination environment. Many MOF structures have poor thermal stability, which leads to the collapse of the framework structure of MOF structures during calcination. Therefore, it is necessary to grasp the reaction conditions. Therefore, the design of nano/microscale MOFs composite structure with high conductivity can maximize the simplification of the preparation process and cost is also an urgent problem to be solved.

Despite the enormous challenges, synthesis of nano/microscale‐MOFs represents a promising approach to achieve the desired function of the material based on rational design. There are still plenty of opportunities for exploration in areas such as catalysis, energy storage, and biomedicine, bringing about novel efficient and facile strategies for challenges in energy, the environment, and human health. We believe that this review will be useful in providing insights into nano/microscale‐MOFs materials and will provide new vision for the future design and application of nano/microscale‐MOFs materials.

## Conflict of Interest

The authors declare no conflict of interest.
